# Targeting retinoic acid receptor alpha-corepressor interaction activates chaperone-mediated autophagy and protects against retinal degeneration

**DOI:** 10.1038/s41467-022-31869-1

**Published:** 2022-07-21

**Authors:** Raquel Gomez-Sintes, Qisheng Xin, Juan Ignacio Jimenez-Loygorri, Mericka McCabe, Antonio Diaz, Thomas P. Garner, Xiomaris M. Cotto-Rios, Yang Wu, Shuxian Dong, Cara A. Reynolds, Bindi Patel, Pedro de la Villa, Fernando Macian, Patricia Boya, Evripidis Gavathiotis, Ana Maria Cuervo

**Affiliations:** 1 Department of Developmental and Molecular Biology, Albert Einstein College of Medicine, Bronx, NY 10461 USA; 2 Institute for Aging Studies of the Department of Medicine of the Albert Einstein College of Medicine, Bronx, NY 10461 USA; 3 Department of Cellular and Molecular Biology, Centro de Investigaciones Biológicas Margarita Salas, CSIC, Madrid, 28040 Spain; 4 Department of Biochemistry, Albert Einstein College of Medicine, Bronx, NY 10461 USA; 5 Department of Pathology, Albert Einstein College of Medicine, Bronx, NY 10461 USA; 6 Department; of System Biology, Universidad de Alcalá, Madrid, Spain and Instituto Ramón y Cajal de Investigación Sanitaria (IRYCIS), Madrid, 28801 Spain; 7 Department of Medicine, Albert Einstein College of Medicine, Bronx, NY USA

**Keywords:** Chaperone-mediated autophagy, Mechanism of action, Retinal diseases

## Abstract

Chaperone-mediated autophagy activity, essential in the cellular defense against proteotoxicity, declines with age, and preventing this decline in experimental genetic models has proven beneficial. Here, we have identified the mechanism of action of selective chaperone-mediated autophagy activators previously developed by our group and have leveraged that information to generate orally bioavailable chaperone-mediated autophagy activators with favorable brain exposure. Chaperone-mediated autophagy activating molecules stabilize the interaction between retinoic acid receptor alpha - a known endogenous inhibitor of chaperone-mediated autophagy - and its co-repressor, nuclear receptor corepressor 1, resulting in changes of a discrete subset of the retinoic acid receptor alpha transcriptional program that leads to selective chaperone-mediated autophagy activation. Chaperone-mediated autophagy activators molecules activate this pathway in vivo and ameliorate retinal degeneration in a *retinitis pigmentosa* mouse model. Our findings reveal a mechanism for pharmacological targeting of chaperone-mediated autophagy activation and suggest a therapeutic strategy for retinal degeneration.

## Abstract (web summary)

Gomez-Sintes et al. have developed small molecules that selectively activate chaperone-mediated autophagy by stabilizing the interaction between retinoic acid receptor alpha and its co-repressor N-CoR1. They demonstrate the protective effect of boosting chaperone-mediated autophagy against retinal degeneration.

## Introduction

Maintenance of proteostasis is essential for normal cellular function and for adaptation to the always changing extracellular environment^1,2^. Chaperones and the proteolytic systems are the major components of the proteostasis network. A gradual loss in functionality of some of these proteostasis pathways with age has been proposed to accelerate the course of degenerative conditions that afflict elders^3^. We have shown that chaperone-mediated autophagy (CMA), a selective mechanism for degradation of cytosolic proteins in lysosomes, declines with age in most tissues from rodents and humans^4^. Furthermore, CMA is vulnerable to the toxic effect of pathogenic proteins that accumulate in neurodegenerative diseases such as Parkinson’s disease or tauopathies^5^. Inhibition of CMA in these conditions further contributes to proteotoxicity in the affected tissues and perpetuates proteostasis failure.

Lower CMA activity in aging mainly results from reduced levels of the lysosome-associated membrane protein type 2 A, LAMP2A (L2A)^6^, the receptor for the substrate proteins delivered to lysosomes by the chaperone Hsc70. The binding of the substrate to the receptor, the limiting step in CMA, triggers L2A assembly into a multimeric translocation complex used by the substrates to reach the lysosomal lumen for degradation^7^. Preventing the decline of CMA in old rodents through genetic manipulations (L2A overexpression) has proven effective in maintaining organ function^8^. Genetic L2A upregulation is also protective in models of Parkinson’s disease-related neuronal toxicity^9^. Thus, CMA activation may be beneficial in diseases where its inhibition has a pathogenic role^5^. CMA has proven to be central to proteostasis maintenance in the retina and to become the main defense against proteotoxic insults with aging, as the other types of autophagy start to fail^10^.

In previous studies, we identified that CMA is under the negative regulation of signaling through the retinoic acid receptor alpha (RARα) and developed first-in-class small molecules capable of upregulating CMA in vitro by blocking the RARα-mediated inhibition on this type of autophagy^11^. Common inhibitors and antagonists of RARα, although also effective in activating CMA, have a negative impact on other types of autophagy such as macroautophagy, since RARα is an activator of this pathway. However, using a structure-based chemical design, we generated small molecules that selectively activate CMA without affecting other forms of autophagy^11^. Although the mechanism behind this selectivity for CMA remains unknown, comparative structural analysis and molecular dynamics suggested that CMA selectivity may be related to the preference of these small molecules to bind and stabilize the open H12 conformation of the RARα ligand-binding domain, which favors the recruitment of corepressors. The small molecules are predicted to use a non-canonical binding mode compared to other RARα antagonists and agonists that commonly use a carboxyl group to form electrostatic interactions with the RARα ligand binding domain^11^.

In this work, we use structure-based drug design to further improve the potency of the original CMA activators and medicinal chemistry optimization to make them suitable for in vivo use. The derivatives demonstrate good biodistribution and pharmacokinetic properties favorable for peripheral and central nervous system targeting. We have found that these compounds stabilize the interaction of RARα with its corepressor N-CoR1. This unique mechanism of action of the CMA activators leads to the selective regulation of only a discrete subset of the RARα transcriptional program, thus conferring selectivity for CMA. We provide evidence that the compounds efficiently activate CMA in vivo without noticeable toxicity. Lastly, taking into consideration the important contribution of CMA to retinal proteostasis^10^, we have used an experimental mouse model of retinal degeneration of clinical relevance for *retinitis pigmentosa*, an incurable devastating condition that results in blindness, and demonstrate that in vivo administration of the CMA activators, either systemically or locally through intravitreal injection, efficiently reduces retinal degeneration and preserves visual function. This work provides proof of concept for pharmacologically targeting the transcriptional mechanism of CMA regulation in a retinal degenerative setting.

## Results

### Small molecules targeting RARα promote CMA activation

**Fig. 1:**
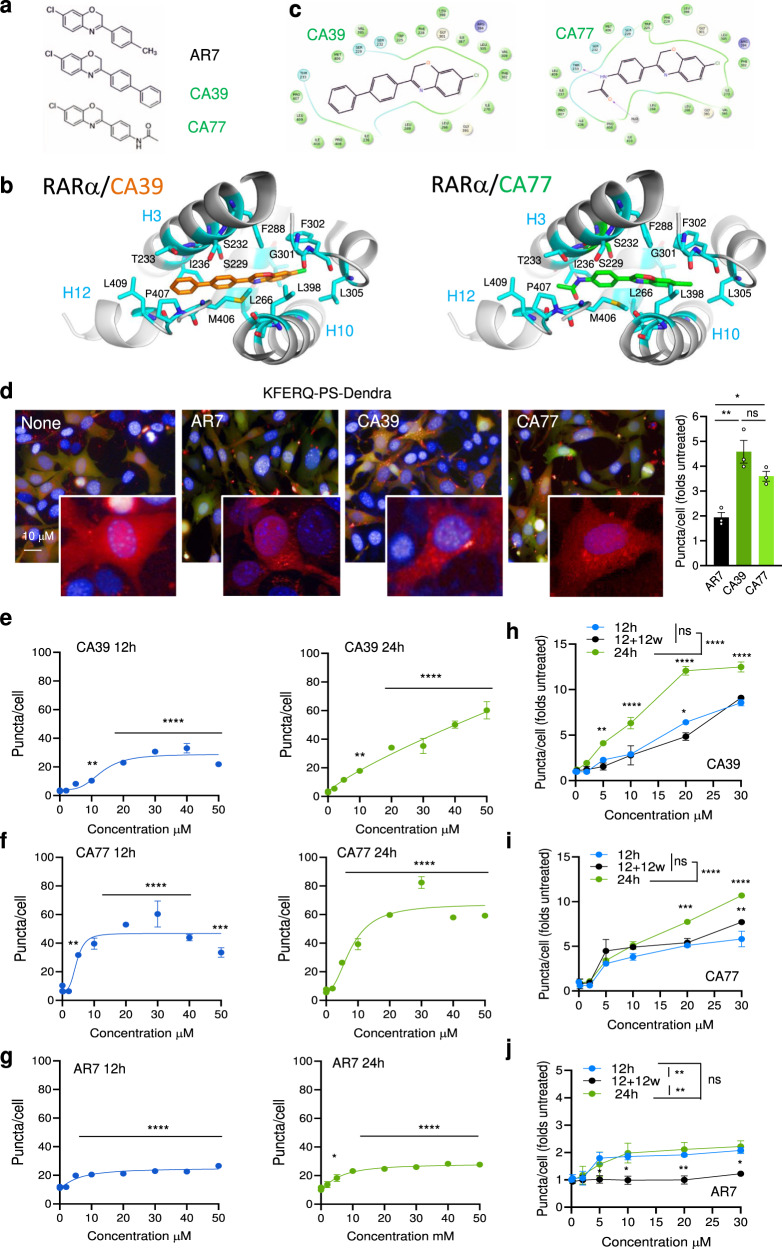
CA39 and CA77 activate CMA in a dose-dependent manner. **a** Chemical structure of AR7, CA39, and CA77. **b** Molecular docking of CA39 (orange sticks) and CA77 (green sticks) in the binding pocket of inactive RARα (PDB ID: 1DKF). **c** Interaction map showing predicted RARα amino acid interactions with CA39 and CA77. **d** CMA activity in NIH3T3 stably expressing the KFERQ-PS-Dendra after incubation with AR7, CA39, or CA77. Representative images of cells treated with 20 μM of each compound. Nuclei are highlighted with DAPI. Inserts shows higher magnification of the red channel. Right shows the comparison with activation of CMA by AR7. n = 3 independent experiments (>1,500 cells counted)**. e**–**g** Quantification of CMA activity in the same cells upon addition of increasing concentrations of CA39 (**e**), CA77 (**f**) or AR7 (**g**) for 12 (left) or 24 (right) hours. *n* = 4 independent experiments (>2,500 cells counted). **h**–**j** Quantification of CMA activity in the same cells after addition of increasing concentrations of CA39 (**h**), CA77 (**i**) or AR7 (**j**) for 12, 24 h, and 12 h after washing (w) them out from the media. *n* = 3 independent experiments (>1,500 cells counted). All values are mean + s.e.m. One-way ANOVA (**d**–**g**) or two-way ANOVA (**h**, **j**) followed by Bonferroni’s multiple comparisons post-hoc test were used. Significant differences with untreated samples are indicated in **e** and among the different incubation protocols in **f**. **p < 0.01, ***p < 0.001, ****p < 0.0001. ns: not significant. Source data and exact *p* values are provided as a Source Data file.

Previously, we generated and validated the AR7 compound as a small molecule binding to RARα ligand-binding domain (LBD) (Fig. 1a) capable of selective activation of CMA in vitro^11^. We have now used an iterative structure-based drug design and medicinal chemistry approach to improve AR7 scaffold for increased CMA potency and pharmacological properties suitable for in vivo use (Supplementary Fig. 1a, b). Analogues (CMA activators; CAs) were evaluated primarily using a fluorescent CMA reporter (see quantification details in Methods) in mouse fibroblasts for displaying more potent activation of basal CMA (cells in serum-supplemented media) and inducible CMA (cells in serum free-media for 16 h)^12^. CA39 and CA77 (Fig. 1a–d) were among the most potent activators of CMA ( >40% CMA of AR7), without noticeable toxicity and able to upregulate both basal and inducible CMA.

Molecular docking studies of CA39 and CA77 consistently favored binding in the RARα-binding pocket formed by the junction of helices h3, h10, and h12 stabilizing the h12 in the open conformation that regulates recruitment of co-repressors and co-activators to RARα, similarly to AR7 (Fig. 1b and Supplementary Fig. 1a). CA39 and CA77 were designed based on the benzoxazine scaffold to increase hydrophobic interactions or polar interactions with the RARα pocket residues respectively. Indeed, both compounds are capable to form extensive hydrophobic contacts and CA77 hydrogen bonding between its amide group with Thr233 and a water molecule near the Pro404 (Fig. 1b, c).

We confirmed that CA39 and CA77 activate CMA in a time- and dose-dependent manner (Fig. 1e, f) with higher potency than AR7 (Fig. 1g). Also, in contrast to AR7, activation of CMA by CA39 and CA77 was still noticeable 12 h after washing out the compounds from the media (Fig. 1h–j). Both compounds efficiently activated CMA in other mouse cell types including neuron-related cells and in human cells where the activating effect of AR7 was very discrete (Supplementary Fig. 2a–c). Interestingly, the activation of CMA elicited by CA77 in the neuron-related cells persisted and even further increased after the compound was removed from the media, suggesting a more robust and prolonged activating effect on these cells.

CA39 and CA77 induced the expected increase in intracellular rates of degradation of long-lived proteins associated with CMA upregulation, but in contrast with AR7, for which the increase in protein degradation was sustained only for the first 12 h after the addition of the compound, protein degradation remained upregulated 24 h after adding CA39 and CA77 (Supplementary Fig. 3a, b). We also confirmed that, as was the case for AR7, CA39 or CA77 did not have the inhibitory effect on macroautophagy, previously described for typical RARα antagonists^11^. In fact, after the addition of any of the three compounds, we did not find significant differences in the percentage of protein degradation dependent on macroautophagy (sensitive to the macroautophagy inhibitor MRT; Supplementary Fig. 3c), the rate of lysosomal degradation of LC3-II (Supplementary Fig. 3d) or the number of autophagosomes and autolysosomes detected using the fluorescent tandem reporter mCherry-GFP-LC3 (Supplementary Fig. 3e). Thus, CA39 and CA77 are more potent CMA activators than AR7 (the hit compound from the previous study^11^) while still preserving their selectivity for this autophagic pathway.

### CA modulate a subset of the RARα transcriptional program

**Fig. 2:**
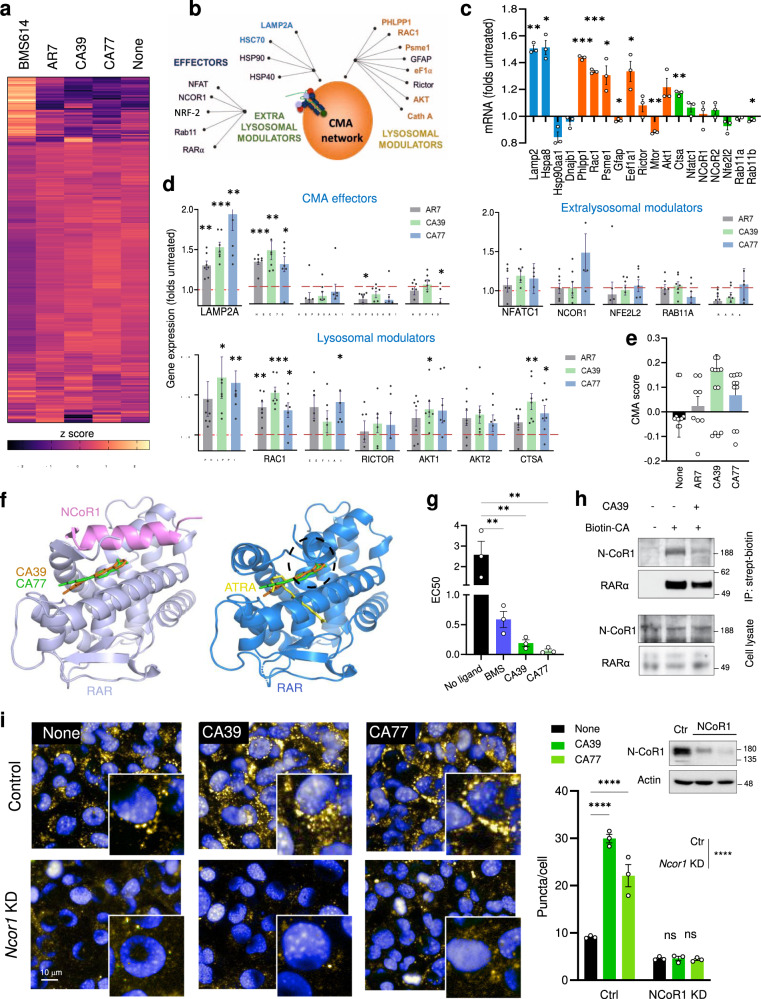
CA induce discrete transcriptional changes by promoting the interaction of RARα and N-CoR1. **a** Heat map of the transcriptional changes in NIH3T3 cells cultured without additions (None) or with 20μM AR7, CA39, CA77, or BMS614 for 24 h. **b**–**d** Effect of CA in components of the CMA network^13^ (**b**). Changing components highlighted in bold. **c** AR7-induced changes in expression of CMA network components. *n* = 3 different experiments. **d**, **e** mRNA levels of the CMA network components by qPCR (**d**) and calculated CMA activation score (**e**) in cells treated as in **a**. *n* = 7 independent experiments. **f** Molecular docking of CA39 (orange) and CA77 (green) is compatible within the inactive (left) conformation of RARα (lilac) bound to N-CoR1 peptide (pink) (PDB ID: 3KMZ). RARa active (blue) conformation is shown for comparison. ATRA (yellow) binds only to the active conformation. Hypothetical binding poses of CA39 (orange) and CA77 (green) are not compatible within the active conformation of RARα due to steric clash (black dashed circle). **g** EC50 (μM) in fluorescence polarization assays with RARα and the N-CoR1 peptide incubated without additions (no ligand) or in the presence of 10 μΜ of BMS394 (BMS), CA39, and CA77. *n* = 3 experiments. **h** Immunoblot for N-CoR1 and RARα of streptavidin pulldowns (top) or total cellular lysates (bottom) of NIH3T3 incubated without additions or with CA39 (10 μM) or biotin-CA (10 μM) for 24 h. IP: immunoprecipitation. This experiment was repeated 3 times. **i** CMA activity in NIH3T3 cells control (empty vector) or knock-down (KD) for N-CoR1 incubated without additions (none) or with 20 μM CA39 or CA77 for 24 h. Left: representative images. Nuclei are highlighted with DAPI. Inserts: higher magnification. Right: Quantification of the number of puncta per cell. *n* = 3 different experiments (>2,500 cells counted). Inset: immunoblot for N-CoR1. Individual values and mean + s.e.m are shown. One sample *t* and Wilcoxon test was used in **c** and **d** and one-way ANOVA (**g**) or two-way ANOVA (**i**) followed by Bonferroni’s multiple comparisons post-hoc test. *p < 0.05, **p < 0.01, ***p < 0.001 and ****p < 0.0001. Uncropped blots are in Supplementary Fig. 10. Source data and exact *p* values are provided as a Source Data file.

To elucidate the mechanism and basis for the selectivity of activation of CMA by AR7, CA39, and CA77 molecules, we performed comparative transcriptomic analysis of cells treated with BMS614, a selective RARα antagonist, with the original AR7 molecule or with CA39 or CA77 (Fig. 2a). In contrast with the marked changes in the transcriptome of cells treated with BMS614, treatment with the CA compounds only induced changes in the expression of a discrete fraction of genes including 8 of the 17 currently accepted components of the CMA network^13^ (Fig. 2b–d). Quantitative qPCR for the 17 CMA components confirmed changes in expression upon addition of the CA compounds, manifested as upregulation of specific CMA effectors and lysosomal modulators (Fig. 2d). This analysis also demonstrated differences in the magnitude of the changes in gene expression between AR7 and the CA (CA39 and CA77) that could explain the higher CMA activation capacity described for the latter in the previous section. In fact, we have recently demonstrated that differences in CMA activity can be inferred by analyzing the expression of the subset of genes that participate in CMA and calculating a CMA activation score (by adding weight and directionality to each of the components in the CMA network)^14^. Using the observed CA-induced transcriptional changes, we found that the predicted CMA score was higher for CA39 and CA77 when compared to AR7 (Fig. 2e). We did not find significant upregulation in the expression of effectors and regulators of macroautophagy and the lysosomal system (CLEAR network; selected genes shown in Supplementary Fig. 4a).

Comparison of the transcriptional profile of cells treated with AR, CA39, and CA77 revealed that beyond the 8 CMA-related genes, only 26 additional genes (11 coding and the rest non-coding or antisense) were modulated by the compounds (14 were changing with the three compounds and 12 of them changed for two of the compounds and revealed a similar trend in the third one) (Supplementary Fig. 4b–d). Some of these proteins and non-coding RNAs could be yet unknown CMA effectors/regulators, as gene set enrichment and node expansion analysis (using STRING database) placed them on pathways that could interact with CMA such as protein and vesicular trafficking, vesicular fusion and ATP generation, and all of them are involved in the cellular response to stress (Supplementary Fig. 4e, f).

Overall, our data supports that, as predicted, CA molecules affect only a very specific subset of RARα regulated genes in which the components of the CMA network are highly represented. These findings highlight major differences between conventional RARα antagonists and the CA compounds and point toward a very different mechanism of action.

### CA stabilize binding of N-CoR1 and RARα

The discrete transcriptional impact of CA compounds and their ability to stabilize RARα in an inactive conformation^11^ made us to consider that they may enhance the interaction of co-repressor molecules with RARα such as N-CoR1^15^. A selective effect of the CA compounds on the co-repressor/receptor interaction could thus explain changes in only a very small subset of RARα -regulated genes. Docking to the N-CoR1 bound RARα crystal structure suggested that compounds’ binding pose is compatible with N-CoR1 binding to RARα but incompatible with the active RARα conformation that cannot bind N-CoR1 or the CA compounds due to steric clashes (Figs. 1b, 2f). Indeed, CA39 and CA77 enhanced N-CoR1 peptide binding to RARα in fluorescence polarization assays (Fig. 2g) and cellular RARα and N-CoR1 could be co-immunoprecipitated after interaction with a biotinylated CA compound (Fig. 2h and Supplementary Fig. 1c), consistent with the docking and binding data. Knock-down of *NCOR1* significantly decreased CMA activity and completely ablated the activating effect of CA39 and CA77 on CMA (Fig. 2i), thus supporting that the effect of the CA compounds is N-CoR1-dependent.

We conclude that the selectivity of CA compounds for CMA stems from their ability to stabilize RARα interaction with co-repressor N-CoR1 and therefore preventing RARα from adopting its active conformation, which requires binding of ATRA substrate and recruitment of co-activators.

### CA compounds activate CMA in vivo

CA39 and CA77 have drug-like properties with reasonable solubility, high to intermediate metabolic stability with human liver microsomes and CA77 is also highly membrane permeable (Supplementary Fig. 5a). ADME properties prediction by Qikprop scores both CAs positive for CNS activity, oral bioavailability, permeability, and unlikely to show HERG K + channel blocking activities (Supplementary Fig. 5b).

**Fig. 3:**
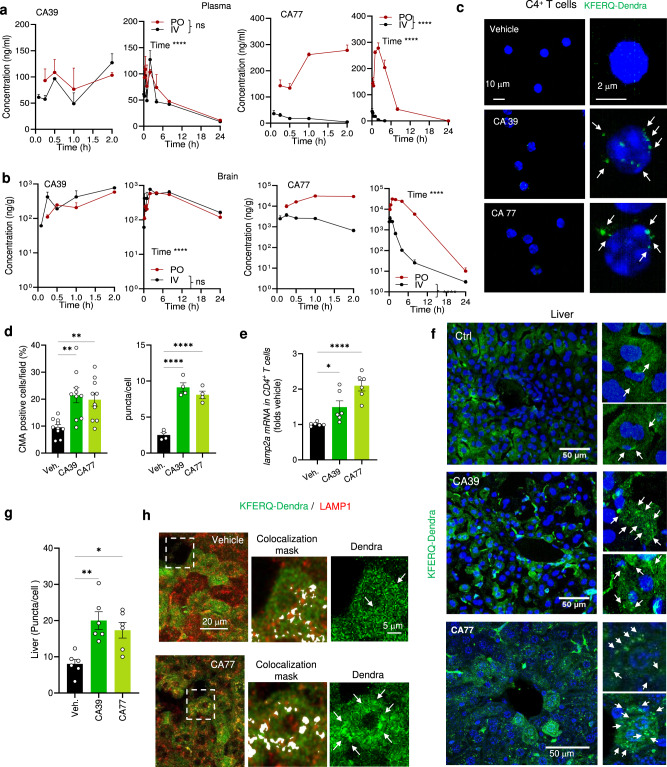
CA compounds activate CMA in peripheral tissues in vivo. **a**, **b** Levels of CA39 and CA77 at the indicated times in plasma (**a**) or brain (**b**) after p.o. (oral, 30 mg/kg bw) and i.v. (intravenous, 1 mg/kg bw) administration in mice. Graph show early (left) and late (right) time points. *n* = 3 mice per time point. **c** Direct fluorescence in CD4 + T cells isolated from blood from KFERQ-Dendra mice i.p. injected daily with (30 mg/kg bw) CA39 or CA77 for three consecutive days. Nuclei are highlighted in blue by DAPI. Right: higher magnification images. Arrows: puncta. **d** Percentage of CD4 + T cells with CMA puncta >3 per cell (CMA + ). *n* = 5 mice per condition. **e** mRNA levels of *lamp2a* in CD4 + T cells activated for 24 h in the presence of CA39 or CA77 (10 μM). Values are expressed relative to untreated cells (None) after normalization by the housekeeping gene actin. Biological triplicates from 2 independent experiments. **f** Representative images of livers from KFERQ-Dendra mice i.p. injected with vehicle, CA39 or CA77 as in **c**. Nuclei are highlighted in blue by DAPI. Insets: higher magnification of sections. Arrows: Dendra^+^ puncta. **g** Quantification of the average number of puncta per cell in liver. *n* = 12 sections from 4 different mice. **h** Representative images of liver sections from mice treated as in c with vehicle or CA77 and co-stained with LAMP1. Merged channels and higher magnification inset of merged with colocalization mask or Dendra fluorescence channel are shown. Arrows: coincidence of fluorophores in puncta. All values are mean + s.e.m. Two-way ANOVA followed by Sidak‘s multiple comparisons post-hoc test was used in (**a**, **b**), and one-way ANOVA followed by Bonferroni’s multiple comparisons post-hoc test in **d, e**, and **g** *p < 0.05, **p < 0.01 and ***p < 0.001 and ****p < 0.0001. ns: not significant. Source data and exact *p* values are provided as a Source Data file.

In vivo pharmacokinetics (PK) studies by intravenous (IV) administration of 1 mg/kg and oral administration (PO) of 30 mg/kg, showed that CA39 and CA77 have good biodistribution. Half-lives in plasma when administered by PO were 7.5 h and 2.2 h for CA39 and CA77, respectively and 8.2 h and 0.6 h for CA39 and CA77, respectively when administered by IV (Fig. 3a and Supplementary Fig. 6a). Both molecules efficiently crossed the blood-brain barrier and displayed significant brain penetrance particularly for CA77, with half-lives in brain of 8 h by IV and 10 h by PO for CA39 and 1.8 h by IV and 4.2 h by PO for CA77 (Fig. 3b and Supplementary Fig. 6b). Their high brain to plasma ratio (Supplementary Fig. 6c) and lack of peripheral blood or major organ (liver, lung, kidney) toxicity in mice upon chronic (5 months) daily oral administration of a more stable CA77 derivative (CA77.1; Plasma half-life 3 h and AUC brain/plasma 5.73 when administered orally^14^) (Supplementary Fig. 7), support that these compounds are suitable lead compounds for targeting central nervous system (CNS) chronic diseases.

We used a transgenic mouse model with the systemic expression of the CMA reporter (KFERQ-Dendra mice)^16^ that allows visualizing CMA activity as a change in fluorescence distribution from a diffuse cytosolic pattern to fluorescent puncta, to explore the ability of CA39 and CA77 to activate CMA in vivo. Considering the pharmacokinetic properties of CA39 and CA77 (Fig. 3a, b, and Supplementary Fig. 6b), we converted Cmax and AUC concentrations from ng/g to μM for each compound and determined that intraperitoneal administration of 30 mg/kg bw of these compounds reaches brain concentrations proven effective in activating CMA in a variety of cell types in vitro (Fig. 1 and Supplementary Fig. 2). Thus, considering either Cmax or AUC the predicted brain concentration of CA77 is in the range of 5-20 μM and of CA39 is 10–20 μM when considering its AUC and extended half-life.

**Fig. 4:**
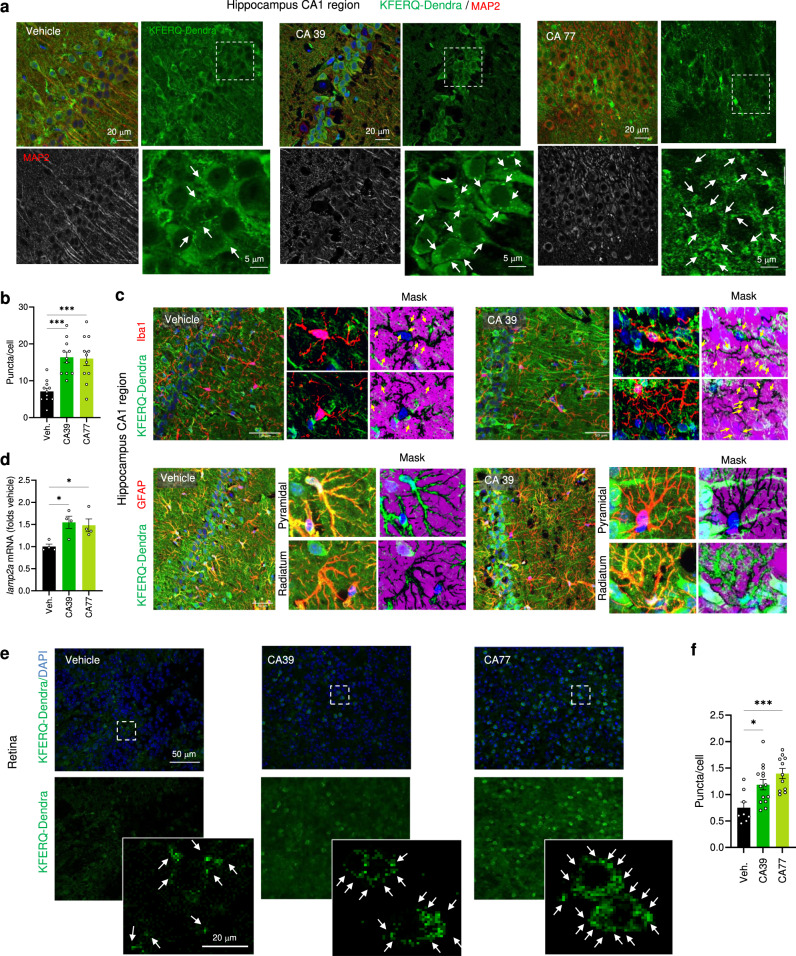
CA compounds activate CMA in CNS and retina in vivo. **a** Representative images of the hippocampus (CA1 region) from KFERQ-Dendra mice i.p. injected with vehicle, CA39 or CA77 daily with (30 mg/kg bw) for three consecutive days co-stained with MAP2. Merged and individual channels are shown. Inset: higher magnification of the boxed area. Arrows: Dendra+ puncta. **b** Quantification of the number of Dendra+ puncta per MAP2 + cell. *n* = 12 sections from 4 different mice. **c** Representative images of the hippocampus (CA1 region) from KFERQ-Dendra mice i.p. injected with vehicle or CA39 as in a co-stained with Iba1 (top) or GFAP/S100 (bottom). Merged channels are shown. Insets: higher magnification to highlight individual cells (left) and inverted mask for the Iba1 (top) or GFAP (bottom) channel to better appreciate Dendra+ puncta. **d** mRNA levels of *lamp2a* in the same brain regions as in **a**. *n* = 4 mice per condition. **e** Representative images of flat-mounted retinas from KFERQ-Dendra mice treated as in **c**. Nuclei are highlighted with DAPI on the top. Insets: higher magnification images. Arrows: Dendra+ puncta. **f** Quantification of the number of Dendra+ puncta per cell. *n* = 14 fields from 3 independent experiments. All values are mean + s.e.m. One-way ANOVA followed by Bonferroni’s multiple comparisons posthoc test was used in **b**, **d**, **f**. *p < 0.05 and ***p < 0.001. Source data and exact *p* values are provided as a Source Data file.

Image analysis of tissues from KFERQ-Dendra mice administered these concentrations daily for 3 days, revealed that both CA39 and CA77 activate CMA with comparable potency in vivo both in peripheral blood cells and in multiple organs. CMA upregulation and increasing *lamp2a* mRNA levels were detected in isolated CD4^+^ T cells of the treated mice, already at 24 h after treatment, providing a convenient method to test for target engagement during the administration of CMA activating drugs (Fig. 3c–e). Imaging of matching hepatic regions of KFERQ-Dendra mice treated with the CA compounds demonstrate a reduction in the cytosolic fluorescent staining and the significant increase in the number of fluorescent Dendra^+^ puncta confirming that both CA39 and CA77 activated CMA in this organ (Fig. 3f–h; colocalization with LAMP1 is shown in h to confirm the lysosomal nature of the of Dendra^+^ puncta). Systemic administration of CA39 and CA77 also upregulated CMA in the brain (Fig. 3a–d). Co-staining with markers for different cell types in the hippocampus demonstrated a significant increase in CMA in neurons (positive for MAP2) as well as upregulation of CMA in microglia (positive for Iba1) (Fig. 4a–c and Supplementary Fig. 8a; high magnification and inverted masks were used to better appreciate the presence of fluorescent puncta in dendrites). In contrast with most peripheric tissues, where at the time of tissue collection the drug-induced transcriptional changes were no longer detected, we could still detect an overall significant increase in *lamp2a* mRNA levels in the hippocampus of CA-treated mice (Fig. 4d). We also confirmed CA-mediated upregulation of CMA in astrocytes from the stratum radiatum layer of the hippocampus, but not in those in the pyramidal layer (Fig. 4c). Same heterogeneity was observed for dopaminergic neurons (TH-positive) in the midbrain substantia nigra region whereby, although there was a significant overall increase in CMA activity in all TH-positive neurons, this upregulation was more noticeable in the smaller subpopulation (Supplementary Fig. 8b–d). Future studies will be needed to determine whether the differences in response are a consequence of the endogenous N-CoR1/RARα levels in these cells or of the natural cross-communication among brain cell types. Systemic administration of CAs was also effective in upregulating CMA in the retina, with remarkable activation of this pathway in the cells of the outer nuclear layer of the retina (rods and cones) (Fig. 4e, f).

### CA have a cytoprotective effect against oxidative stress

CMA is upregulated in response to oxidative stress^17^ and has been shown to be protective against this insult both in vitro and in vivo^8,18^. The beneficial effect of activation of CMA under these conditions is a combination of its ability to selectively eliminate oxidized proteins through lysosomal degradation and of adjusting the cellular metabolic activity to reduce free radical production^19,20^. We used oxidative stress paradigms in cultured cells and tissue explants to test the cytoprotective effect of the CA compounds in these settings.

**Fig. 5:**
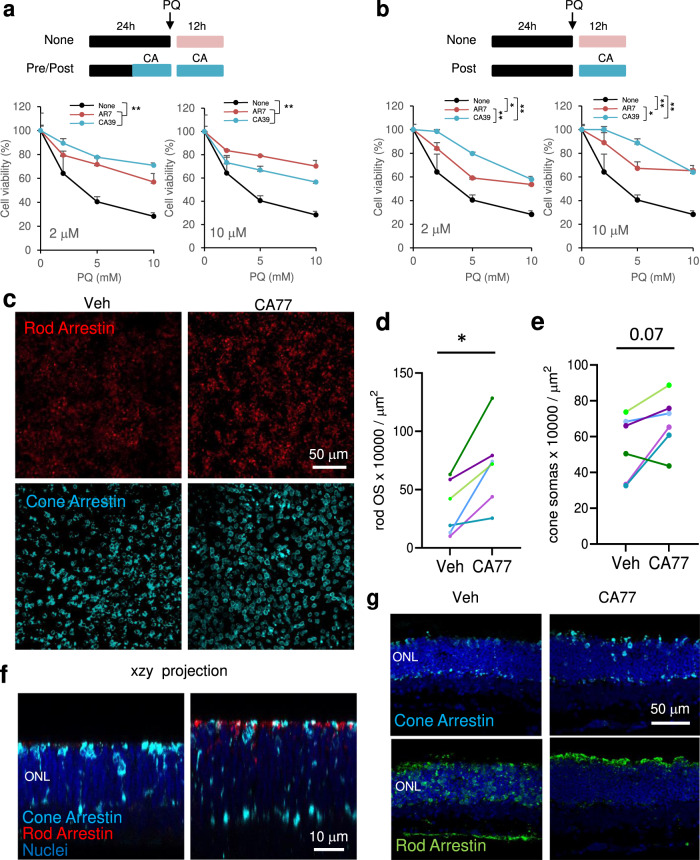
CA compounds are cytoprotective in cells and tissues. **a** Cell viability of NIH3T3 exposed to the indicated concentrations of paraquat (PQ) after 12 h treatment with 2 μM (left) or 10 μM (right) the indicated compounds. The treatment with PQ alone (None) or in the presence of the compounds lasted 12 h. *n* = 3 independent experiments. **b** Cell viability of NIH3T3 simultaneously exposed to the indicated concentrations of paraquat (PQ) and 2 μM (left) or 10 μM (right) of the indicated compounds for 12 h. *n* = 3 independent experiments. **c** Immunostaining for the indicated markers of rods and cones in whole-mount retinas from rd10 mice maintained without additions (right eye; Vehicle (Veh.)) or in the presence of CA77 (left eye) for 72 h. Right: Quantification of the OS stained for rod arrestin (**d**) or number of cone arrestin-positive somas per field (**e**) in retinas from the right and left eye of each animal. *n* = 8 mice (representative experiment from 3 independent experiments). All values are mean + s.e.m. **f** Orthogonal xzy projection corresponding to the areas above show from a different angle to better appreciate preserved ONL and rod and cone OS in CA77 compared to vehicle-treated mice. **g** Images showing staining with rod and cone arrestin in standard 12 µm-cryosections show comparable results to those obtained by whole-flat mounts. Two-way ANOVA followed by Bonferroni’s multiple comparisons posthoc test was used in **a** and **b**. Significant differences between treatments are shown in the legend and specific differences between treatment for a given PQ concentration in the figure. Data from the three independent experiments in **d** to compare the effect of vehicle and CA77 on arrestin OS counts or cone arrestin soma numbers, were analyzed by paired two tailed *t* test. *p < 0.05 and **p < 0.01. Source data and exact *p* values are provided as a Source Data file.

We exposed cultured cells to increasing concentrations of the pro-oxidant agent paraquat (PQ) and confirmed that CA39 reduced cell death when administered before the insult (Fig. 5a), as previously shown also for AR7^11^, but it displayed significantly higher protection than AR7 when added after the insult (Fig. 5b).

To test if the protective effect of CA was also noticeable in whole tissues, we used CA77 in retinal explants of rd10 mice, an experimental model of *retinitis pigmentosa*. Rd10 mice harbor a missense mutation in the *Pde6b* gene and display photoreceptor cell death and vision loss^21^. We favored this model over other mouse models of retinitis pigmentosa because it produces a delayed phenotype that allows for the acquisition of functional data (electroretinograms) before degeneration, and where vision loss better resembles the course of the disease in patients^22^. Administration of CA77 to whole retinal explants obtained from rd10 mice from P21 to P24, led to significant preservation of the number of rods (rod arrestin-positive), which are the structures initially affected in the disease, and also to higher preservation for cones (cone arrestin-positive) when compared to the contralateral untreated retinal explant (Fig. 5c–e**;** note that the sparse staining for rod arrestin is a result of the almost complete loss of the outer segments (OS) at this postnatal time). Preservation of the outer nuclear layer (ONL) in the CA77-treated retinas was observed in the xzy orthogonal projections from the retinal flat mounts (Fig. 5f) or upon staining with the same markers of 12 µm cryosections from the same retinal explants (Fig. 5g). More importantly, the outer segments of rods and cones were better preserved in explants cultured for 72 h with CA77 (Fig. 5g). These results support improved cytoprotective effect of the CMA activators reported in this work and demonstrate their ability to function in whole tissues.

### CA ameliorates retinal degeneration

**Fig. 6:**
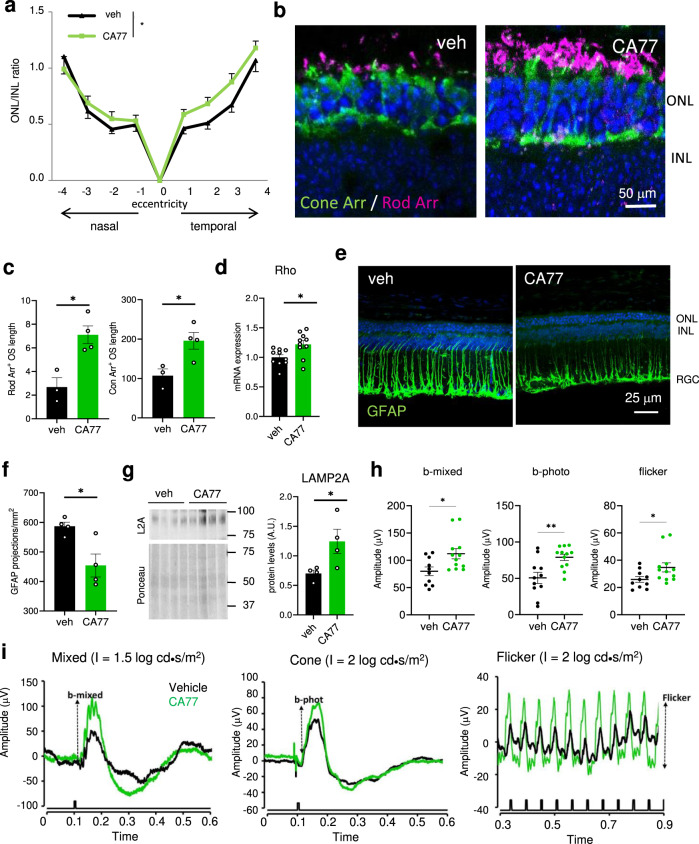
CA compounds prevent rd10 retinas degeneration. **a** Ratio of the thickness of the outer nuclear layer (ONL) and inner nuclear layer (INL) in retinas of rd10 mice treated from P18 to P25 with daily i.p. injection of the vehicle only or 40 mg/kg bw of CA77. *n* = 8 (vehicle) and 9 (CA treated), from 3 independent experiments. **b** Cone (green) and rod (magenta) arrestin markers in temporal central retina of rd10 mice treated as in **a**. Nuclei are highlighted with DAPI. **c** Quantification of outer segment (OS) length of the rods (left) and number of cones (right) measured in the whole retina with the markers used in **b**. *n* = 4 areas per animal, 4 mice per condition. **d** mRNA levels of *rho* in the same animals used in **a**. *n* = 10. **e**, **f** Representative image of the immunostaining for GFAP in the same retinas (**e**) and corresponding quantification of the GFAP projections (**f**). *n* = 4. **g** Immunoblot for the indicated proteins in retinas of mice treated as in **a**. 4 different mice are shown. *Right*: Densitometric quantification of L2A in *n* = 4 different mice. Values are expressed as arbitrary units. **h**, **i** Electroretinogram wave amplitudes at P33 from rd10 mice injected with CA77 or vehicle as in **a** (**h**) measured as shown in the traces (**i**). *n* = 10 veh and 12 CA77. Individual values and mean + s.e.m. are shown. Two-way ANOVA followed by Bonferroni’s multiple comparisons post-hoc test was used in **a**, and unpaired two-tailed *t* test in all others. *p < 0.05 and **p < 0.01. Uncropped blots are in Supplementary Fig. 10. Source data and exact *p* values are provided as a Source Data file.

The favorable in vivo PK properties of CA39 and CA77 (Fig. 3a, b and Supplementary Fig. 6), their ability to activate CMA in whole animals (Figs. 3 and 4) and their remarkable protective effect in the rd10 retinal explants (Fig. 5c–g) motivated us to evaluate their therapeutic potential in the *retinitis pigmentosa* model in vivo (Fig. 6). In this model, photoreceptor loss starts with acute rod cell death that peaks at p22 and it is followed by a more progressive cone cell death^21^. To make the intervention clinically relevant, we initiated the treatment around the peak of maximal rod death (from p18 to p25). We found that retinas from rd10 mice receiving a daily intraperitoneal injection of CA77 (40 mg/kg bw) for one week showed significantly thicker outer nuclear layers (ONL), corresponding to photoreceptor nuclei, indicative of less cell loss (Fig. 6a).

Immunostaining with markers of rods (rod arrestin and transducin) and red and green cones (opsin R/G) or with the pan-cone marker (cone arrestin) also revealed the increased length of the outer segments of photoreceptors in the CA77-treated mice compared with those receiving vehicle (Fig. 6b, c and Supplementary Fig. 9a, b). Higher mRNA levels of photoreceptor-specific proteins (i.e., *rhodopsin* shown in Fig. 6d) in these animals, further confirmed higher cellular preservation. We also compared levels of retinal inflammation as an additional marker of disease progression. As shown in Fig. 6e, f, retinas from CA77-treated mice have strikingly lower levels of GFAP, a well-known marker of astrocyte and activated Müller cells. Also, as in the case of other organs, by the time of tissue collection we did not detect differences in *lamp2A* mRNA levels, immunoblot for L2A demonstrate that the drug was effective in significantly increasing retinal levels of the limiting CMA component, thus confirming target engagement (Fig. 6g).

To confirm that the CA77-mediated improvement in the retinal structure is associated with preserved visual function, we performed electroretinograms (ERG). Mice receiving CA77 from P18 displayed significantly higher amplitude of their mixed (rod and cone) scotopic responses (b-mixed), and cone photopic responses (b-phot and flicker waves) (Fig. 6h, i), which corroborate the beneficial effect of the intervention on retinal function. No significant differences in other light responses measured were observed between mice receiving CA77 or not.

**Fig. 7:**
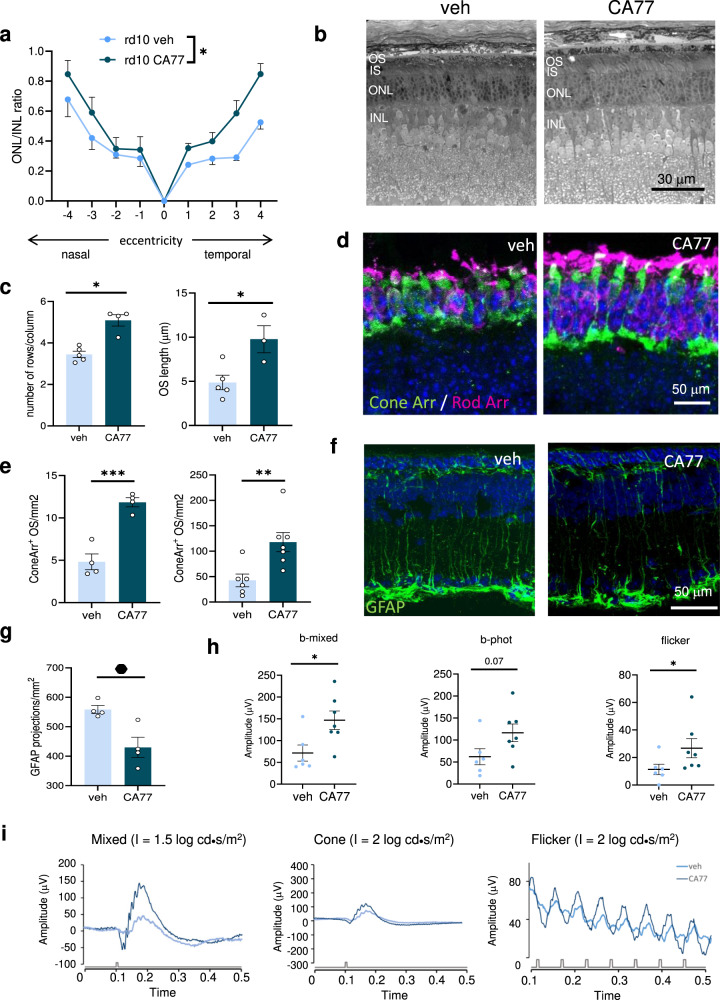
Single intravitreal CA77 injection reproduces neuroprotection obtained with systemic CA77 administration. **a** Spider graphs of the ratio of the thickness of the outer nuclear layer (ONL) and inner nuclear layer (INL) in retinas of rd10 mice 7 days after receiving at P18 a single intravitreal injection of CA77 (40 µM) or vehicle. *n* = 4 (vehicle) and 7 (CA treated), from 3 independent experiments. **b**, **c** Toluidine blue staining in plastic embedded sections from the same animals. Representative images (**b**) and quantification of the number of rows per column (left) and OS length (right) in the semithin sections (**c**) are shown. *n* = 4 animals. **d**, **e** Cone (green) and rod (magenta) staining in cryosections of rd10 mice treated as in **a**. Representative images (**d**) and quantification of rod (left) and cone (right) arrestin in outer segment (OS) (**e**) are shown. Nuclei are highlighted with DAPI. *n* = 4 (for rod arrestin) and 6 (for cone arrestin) mice per condition. **f**, **g** Representative image of the immunostaining for GFAP in the same retinas (**f**) and corresponding quantification of the GFAP projections (**g**). *n* = 4. **h**, **i** Electroretinographic responses of rd10 mice intravitreally injected with CA77 or vehicle. Wave amplitudes (**h**) and representative waves of electroretinograms (**i**) are shown. *n* = 6 veh and 7 CA77. Individual values and mean + s.e.m. are shown. ANOVA of repeated measures was applied to data in **a**. Two-sided unpaired *t*-test was applied in the rest of analysis shown. *p < 0.05, **p < 0.01 and ***p < 0.001. Source data and exact *p* values are provided as a Source Data file.

We next evaluated a more translational approach by delivering the CA compound intravitreally, the preferred delivery route in clinical practice. Analysis of rd10 mice retinas 7 days after a single intravitreal injection of CA77 (40 µM) at P18 revealed thicker retinal ONL/INL ratio (Fig. 7a) and better OS and ONL preservation, both in cryosections and plastic embedded sections (Fig. 7b, c and Supplementary Fig. 9c) in drug-treated compared to vehicle-treated mice. Photoreceptor staining with rod and cone arrestin further corroborated the cytoprotective effect of CA77 in the rd10 mice retinas (Fig. 7d, e). Gliosis was also significatively improved after CA77 administration (Fig. 7f, g and Supplementary Fig. 9d). More importantly, CA77 administration preserved vision in the rd10 animals determined 7 days after the single intravitreal injection (Fig. 7h, i and Supplementary Fig. 9e).

**Fig. 8:**
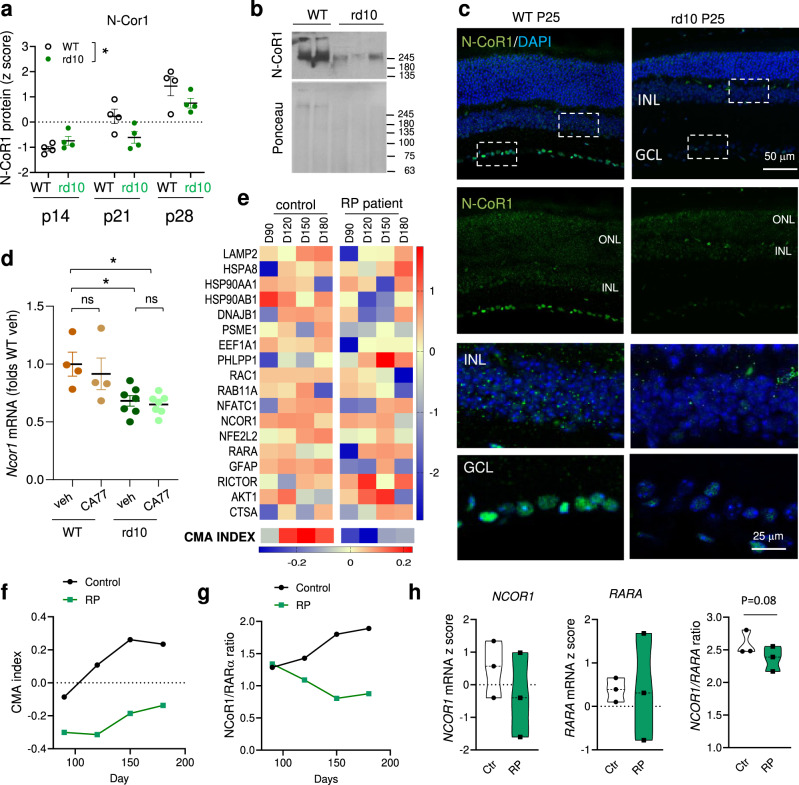
N-CoR1 expression is reduced in *retinitis pigmentosa*. **a** Protein levels of N-CoR1 in retinas from wild type (WT) and rd10 mice at the indicated postnatal (P) days. Data from^23^. Values are expressed as Z score. *n* = 4 mice/genotype/time. **b** Immunoblot for N-CoR1 in retinas of 2 WT and 3 rd10 mice. Ponceau staining is shown as loading control. **c** Immunostaining for N-CoR1 in cyrosections of WT and rd10 mice retinas at p25. Nuclei are highlighted with DAPI. Merged (DAPI and N-CoR1) (top) and single (N-CoR1) (bottom) images. Bottom: Higher magnification of boxed regions from the inner nuclear layer (INL) (top) and in the ganglion cell layer (GCL) (bottom). **d** *Ncor1* mRNA levels in WT and rd10 mice treated from P18 to P25 with daily i.p. injection of vehicle or CA77 (40 mg/kg bw). Values are expressed as folds WT vehicle. *n* = 4 (WT) and 7 (rd10) mice. **e** Heat map of CMA network genes expression in retinal organoids from healthy (Control) and *retinitis pigmentosa* patients (RP) bearing the PDE6B mutation. D: days of organoid differentiation. D90-D180: features of mid-state disease; D230: features of late-state disease. CMA index^14^ is shown at the bottom. Data from^24^. **f**, **g** CMA activation index (**f**) and ratio of *NCOR1* to *RARA* mRNA levels (**g**) in the same samples as in **e**. RNA was isolated from 3-5 organoids from two independent differentiations. **h** mRNA levels of *NCOR1* (left), *RARA* (middle) and *NCOR1* to *RARA* ratio in retinal organoids from Control and RP patients bearing mutations in RP2 at D180. Data from^25^ *n* = 3 individuals per diagnosis (minima, maxima, center, bounds of box, whiskers and percentile, are detailed in the Source Data File). Individual values and mean + s.e.m. are shown. Two-way ANOVA was used in a, One-way ANOVA with Tukey’s multiple comparison post-hoc test in **d** and two-sided paired *t* test in h. *p < 0.05 and **p < 0.01. ns, not significant. Uncropped blots are in Supplementary Fig. 10. Source data and exact *p* values are provided as a Source Data file.

Interestingly, analysis of data from a proteomic study of retinas from rd10 mice at pre-, peak- and post-degeneration time points^23^ revealed a significant decrease in retinal levels of N-CoR1 (Fig. 8a). We also confirmed by immunoblot and immunostaining for N-CoR1 a very pronounced decrease of this protein in rd10 retinas compared to control retinas (Fig. 8b, c). Reduced levels of N-CoR1 in rd10 retinas seem to be primary a result of transcriptional downregulation of this gene (Fig. 8d). As expected from the mechanism of action of CA77 described in this work, the drug did not change the expression levels of *NCOR1* in either of the experimental groups, further supporting that that CAs are able to restore CMA activity by stabilizing N-CoR1/RARα interaction and thus maximizing the effect of the remaining N-CoR1 protein.

Lastly, to investigate the possible translational value of CAs in the human disease, we analyzed the status of CMA-related genes and *NCOR1* expression using data from a previous study in patient-specific retinal organoids shown to recapitulate RP features^24^. Although direct measurement of CMA activity is not possible in human retina, we used the expression components in the CMA network to calculate the CMA score^14^. We found that the CMA activation score increases with human retinal organoid maturation (considered to be fully developed by 150 days), whereas retinal organoids from RP patients with the PDE6B mutation (the same mutation as the rd10 mouse model) displayed a marked reduction in the CMA activation index at all stages (Fig. 8e, f). A pronounced increase in the ratio of co-repressor to receptor (*NCOR1* to *RARA* ratio) weighted heavily in the observed upregulation of CMA in the early stages of maturation in healthy human retinal organoids (Fig. 8e, g, black line). In contrast, reduced *NCOR1/RARA* expression ratio, more noticeable as the retinal organoids reached full maturation, was observed in the RP patient retinal organoids (Fig. 8e, g, green line). We observed a similar trend toward reduced *NCOR1* expression and increased *RARA* and overall reduced *NCOR1/RARA* expression ratio upon analysis of a second study using human iPSC-derived retinal organoids from RP patients bearing instead RP2 mutations, that account for ~5% of all cases of X-linked RP^25^ (Fig. 8h). These findings support that reduced *NCOR1/RARA* expression ratio and the subsequent lower CMA activity may be a common feature in RP patients and that interventions that stabilize the interaction of the co-repressor with the receptor, as the one described in this work, could be successful to restore the normal N-CoR1/RARα tone in the human disease.

## Discussion

In this work, we have developed and characterized two activators of CMA with oral bioavailability, significant brain exposure and favorable drug-like properties suitable for chemical activation of CMA in different tissues in vivo. CMA activators demonstrated their efficacy in ameliorating retinal degeneration and disease progression in a mouse model of *retinitis pigmentosa*. Our findings as part of the characterization of these small molecules support that their ability to selectively activate CMA in vivo, good biodistribution, and low toxicity makes them promising candidates for lead optimization for future drug development.

Our study represents a major advance on our understanding of the mechanism whereby this family of small molecules modulates CMA. Although originally developed against RARα, because of its inhibitory effect on CMA^11^, our earlier structural studies already revealed non-canonical binding positioning of these compounds on RARα very different from that of agonists and antagonists of this receptor. This led us to describe them as non-canonical inhibitors of RARα^11^. In this work, we have unveiled their unique ability to stabilize RARα/N-CoR1 interaction which explains why only a subset of the RARα transcriptional program changes upon addition of CA. Our studies also highlight N-CoR1 as a previously unknown modulator of CMA and link failure of this RARα co-repressor to disease, as exemplified here in the case of *retinitis pigmentosa*. The effective CMA activation by the CA compounds despite the very discrete transcriptional changes imposed by these molecules suggests that N-CoR1, through its binding to RARα, activates expression of key CMA components, in a similar way to TFEB regulating the macroautophagy transcriptional program^26^. Future studies will determine whether some of the products of the 26 coding and non-coding additional genes modulated by the CA compounds also play functional or regulatory roles on CMA. As expected, we found some temporal dissociation on the drug-induced changes at the transcriptional level (which occur first) and those at the protein level and in CMA activity (since CMA activation in this context is attained through de novo synthesis of specific components of the CMA network). However, we noticed some tissue-specific differences in the duration of the transcriptional upregulation, with organs such as in liver or retina where it was no longer detected at the time of tissue harvesting, while others such as brain, where mRNA levels of *lamp2a* were still elevated in the treated group at harvesting. Future studies are needed to clarify if the sustained transcriptional upregulation in the brain is due to the higher concentrations that the compounds reach in this organ, or if it is a result of cell-type differences in the magnitude and duration of the compound-induced transcriptional upregulation.

Increasing evidence support contribution of loss of proteostasis to the pathogenesis of common degenerative diseases^27^. In addition, loss of proteostasis has also been described as one of the pillars of aging^28^, and consequently as an important contributor to the aggravating effect of aging in age-related disorders. Despite growing interest in pharmacological targeting of the proteostasis network with therapeutic purposes, the success of such approaches is still discrete because of lack of selectivity^29^. In the specific case of autophagy, inhibition has also proven easier than selective chemical activation. Although drugs such as rapamycin or natural molecules as spermidine have demonstrated potent macroautophagy activation properties, they lack the selectivity desirable when considering the chronic nature of treatments for age-related disorders^30^. Emphasis is also now placed on development of drugs that can selectively activate a particular type of autophagy without affecting others^5^. Chemical activation of CMA fits well in this shift toward more selective modulation of autophagy.

The availability of compounds with activating effect on CMA is currently very limited and in general they lack selectivity or have only been tested in vitro (reviewed in^31^). CMA upregulation has been described after administration of compounds that modulate glucose metabolism or that block protein translation, but their effect on CMA is indirect^32^. Although several CMA components, such as PHLPP, mTOR, Akt, NFAT, are pharmacologically targetable, their involvement in multiple cellular processes beyond CMA makes chemical refinement of the targeting compounds necessary to confer their selectivity for CMA. A recent study identifying that class I but not class III PI3K inhibitors activated CMA holds promise as a way to prevent simultaneous inhibition of macroautophagy^33^.

Concerns expressed about the possible detrimental effects of persistent upregulation of autophagic pathways, such as macroautophagy, are less critical in the case of CMA. Although CMA degrades both damaged/altered proteins (for quality control) and fully functional proteins (to suppress their biological function for regulatory purposes), all CMA substrates need to be first tagged for degradation. The very first step in CMA is substrate identification by hsc70, consequently, only proteins that have been previously selected for either quality control or regulatory degradation by the chaperone will benefit from enhanced CMA activity. Proteins that are not previously identified for CMA targeting by hsc70 will be spared, independently of how much CMA is upregulated. This safety mechanism explains why overexpression of L2A in old mice markedly reduced the intracellular proteotoxicity characteristic of old tissues, without causing a disbalance of the normal proteome composition^8^. The absence of cellular or tissue damage observed in this study in animals receiving daily doses of CA for 5 months (what approximately equals 15 years in humans) supports that chronic upregulation of CMA has no negative effects. Furthermore, the unique mechanism of action of CA compounds identified in this work also dissipates concerns about possible negative effects of inhibition of RARα, since we have shown that most of the signaling downstream of this important nuclear receptor remains intact.

In light of the growing number of connections linking CMA malfunctioning and disease^4^, we anticipate that molecules such as the CA compounds developed in this study, could have translational value in treatment of an array of degenerative conditions. In this work, we present evidence of their protective effect against retinal degeneration using a pre-clinical model of *retinitis pigmentosa*. Although a rare genetic disorder, the severity of the visual deficiency and rapid progression to blindness, along with the fact that there are no currently effective treatments for this type of retinal degeneration, justify our interest in further exploring the suitability of these CA compounds or additional derivatives in the treatment of this detrimental retinal condition. We have previously shown that CMA has a key role in preserving retinal proteostasis^10^. Here, we wanted to investigate whether the protective effect of CMA in retina extended also to other types of stressors, beyond proteotoxicity. To this effect, we selected the rd10 mouse model of retinitis pigmentosa where degeneration is associated with an abnormal increase in the levels of cGMP that results in photoreceptor cell death. Moreover, and in contrast to other Pde6 mutations that degenerate very fast after the time of eye opening (i.e., rd1 mice), the rd10 mouse model provides a suitable therapeutic window and eliminates the confounding effects of the naturally occurring cell death associated to postnatal retinal development^22^. Using this model, we now show that both systemic as well a single intravitreal injection of CA77 provide strong preservation of retinal histology and visual function. In addition, our observation that *NCOR1* expression was reduced in retinas from RP patients bearing different mutations suggests that dysregulation of the N-CoR1/RARα axis could be a common pathogenic feature in this group of diseases and supports the possible translational value of our CA compounds. Furthermore, the cytoprotective effect of the pharmacological activation of CMA in the retina opens the possibility of expanding testing of the CA compounds to common retinal degenerative disorders such as diabetic retinopathy or age-related macular degeneration.

## Methods

### Animal, cells, and reagents

#### Animals

C57BL/6 J mice wild-type (WT) and homozygous for the *Pde6* mutation (rd10 mouse model of retinal degeneration) were obtained from The Jackson Laboratory and experiments were performed at the age indicated in each part of the study. C57BL/6 KFERQ-Dendra mice were generated by back-crossing FVB KFERQ-Dendra mice with wild-type C57BL/6 mice for 8 generations^16^. Both male and female animals were used in this study in equally distribution groups. However, results from both sexes were pooled, because of absence of significant differences in any of the parameters analyzed after statistical analysis with a mixed model of two-way ANOVA sex and treatment as independent variables and the corresponding measure as dependent variable. All animals were housed in a barrier-controlled facility (19–23 °C 30–60% relative humidity; 12 h light/dark cycle) with *ad libitum* access to standard chow pellets (protein 19 gm%/carbohydrate 67 gm%/fat 4 gm%) and water. For animal administration of CA compounds, a formula containing 30% PEG 400 (Sigma, #8.0745), 65% of 5% glucose solution, 5% Tween 80 (Sigma, #P1754) was used to dissolve them. CA compounds were prepared freshly by adding 5% DMSO and sonicating for 1 h. Treatment of KFERQ-Dendra mice with the CA compounds was done at 4 months of age by i.p. daily injection of 30 mg/kg bw or vehicle for three consecutive days and tissues collected 6 h after the last injection. In the case of rd10 mice, animals were daily injected from P18 to P25 with CA77 (40 mg/kg bw). In the case of intravitreal administration, a single intravitreal injection was applied at P18 with CA77 (40 µM) in DMSO/PBS and the contralateral eye was injected with PBS and DMSO at same concentration. At P26, mice were sacrificed and eyes fixed overnight with 4% PFA (Sigma, #P6148) in PBS at 4 °C for immunofluorescence or retinas dissected and immediately frozen for biochemistry. Distribution of animals in the vehicle or treatment group was done randomly. ICR (CD-1) outbred 3 months old male mice from ENVIGO were used for the pharmacokinetic studies (see pharmacokinetic section for housing and handling details). All animal studies and procedures complied with ethical regulations, were performed in accordance with the European Union guidelines and the ARVO Statement for the Use of Animals in Ophthalmic and Vision Research and were approved by the Institutional Animal Care and Use Committee at the Albert Einstein College of Medicine, the CSIC Bioetica Comite and approved by the Comunidad de Madrid, PROEX232/17.

#### Cells

NIH3T3 mouse fibroblasts (CRL-1658), CaCo2 cells human colorectal adenocarcinoma (HTB-3), and the N2a neuroblastoma cell line (CCL-131) were obtained from the American Type Culture Collection and were validated by genomic PCR. Primary human fibroblasts (GM01651) were from Coriell Repository. Cells were cultured to full confluence in a 37 °C incubator with 5% CO2 in complete Dulbecco’s modified Eagle’s medium (DMEM, Sigma #D5030) supplemented with 10% heat-inactivated newborn calf serum (HyClone #SH30401.02HI), 1% penicillin/streptomycin/fungizone (Invitrogen, #10072322). All the cell lines were tested for mycoplasma contamination using DNA staining protocol with Hoechst 33258 dye and none of them was found positive. Knock-down of *Ncor1* was done using lentiviral mediated shRNA (SHCLNG-NM_011308) from the Sigma Mission library following standard procedures. The efficiency of knock-down was tested by immunoblot. Cell viability was measured using the CellTiter-Blue kit (Promega, #G9241) 24 h after the addition of the different stressors according to the manufacturer’s instructions. All cell lines were validated by genomic PCR.

#### Antibodies

Primary antibodies were from the following sources: (dilution for use in immunoblot (IB) or immunofluorescence (IF) and clone indicated in brackets): rabbit anti LC3B (1/1000 IB, MBL pm036), mouse anti β-actin (1/10000 IB, Sigma, A4700), chicken anti MAP2 (1/2000 IF, Biolegend, 822501), mouse anti-GFAP (1/1000 IF, Millipore, MAB360), rabbit anti-GFAP (1/500 IF, DAKO, Z0334), mouse anti S100 (1/1000 IF, Abcam, ab7852), goat anti Iba1 (1/100 IF, Abcam, ab5076), rabbit anti-RARα (1/1000 IF and IB, Cell Signaling, 2554), mouse anti visual (rod) arrestin (1/200 IF, Santacruz Biotechnologies, C-3, Sc-166383), rabbit anti-Opsin R/G (1/1000 IF, Millipore, AB5405), rabbit anti transducin (1/200 IF, Santacruz Biotechnologies, sc-389), rabbit anti cone arrestin (1/1000 IF, Millipore, AB15282), rabbit anti-N-CoR1 (1/100 IF and IB, Cell Signaling, 5948) and rabbit anti L2A (1/2000 IB, Invitrogen, 51-2200). Secondary antibodies were from the following sources (dilution, source, and catalogue number): Anti-mouse IgG secondary antibody Alexa Fluor 568 (1/500 IF, Invitrogen, A-11004), Anti-mouse IgG secondary antibody Alexa Fluor 647 (1/500 IF, Invitrogen, A-32728), Anti-goat IgG secondary antibody, Alexa Fluor 594 (1/500 IF, Invitrogen, A-11012), Anti-rabbit IgG secondary antibody, Alexa Fluor 568 (1/500 IF Invitrogen, A-11011), Anti-chicken IgY secondary antibody, Alexa Fluor 488 (1/500 IF, Invitrogen, A-11039), Anti-rabbit secondary antibody, HRP (1:5000 IB, ThermoFisher, 31460), Anti-mouse secondary antibody, HRP (1:5000 IB, ThermoFisher, 31430).

All antibodies used in this study were from commercial sources and once received in our laboratories we determine the optimal conditions for our experiments using the multiple dilution method to determine the higher dilution that was giving a signal in order to minimize the detection of unspecific signals. In the case of antibodies against LAMP2A, RARα, and N-CoR1 we used knock-down cell lines for validation of the antibodies.

### Human retinal tissue

Data from human tissue from healthy individuals and *retinitis pigmentosa* patients was obtained from previous published studies and no recruitment or collection of human tissue was performed for the completion of this work. Sources of the human tissues for the generation of the retinal organoids are detailed in the original studies^24,25^. Briefly, in^24^ patient mononuclear cells were collected and subjected to a plasmid-based reprogramming system to generate human iPSCs that were subsequently subjected to protocols for trilineage differentiation. In^25^, consent skin biopsies were obtained to isolate dermal fibroblasts, and iPSCs were generated from two unrelated RP patients and 2 controls. In both studies, collection of human samples was approved by the research ethics committees in their respective institutions (The Eye Hospital of Wenzhou Medical University Ethics Committee^24^ and The Moorfields Eye Hospital and Royal Victoria Eye and Ear Hospital Dublin Research Ethics Committees^25^). The patients/participants provided their written informed consent to participate in those published studies^24,25^.

### Autophagic measurements

#### Intracellular proteolysis of long half-life proteins

Confluent cells were labelled with [^3^H]leucine (2μCi/ml) (NEN-PerkinElmer Life Sciences) for 48 h and then extensively washed and maintained in medium with an excess of unlabeled leucine^34^. Proteolysis was calculated from aliquots of the medium taken at different times and precipitated in trichloroacetic acid, as the amount of acid-precipitable radioactivity transformed to acid-soluble radioactivity at each time. Where indicated cell were treated during the chase with 100 mM leupeptin (Sigma, #108975) and 20 μM NH_4_Cl (to determine lysosomal degradation) or with 100 μm MRT 68921 dihydrochloride (Tocris, #5780) (to determine macroautophagy degradation).

#### Macroautophagy activity

Cells were transduced with a lentiviral vector expressing mCherry-GFP-LC3^35^, fixed and flux determined as the conversion of dual fluorescence puncta (autophagosomes) into only red fluorescent puncta (autolysosomes). Flux was also measured by immunoblot for LC3 in cells incubated for 6 h with 20 mM NH_4_Cl/100 μM leupeptin, by discounting the intensity of LC3 in non-treated cells from that in cells treated with the inhibitors.

#### CMA activity

Cells were transduced with lentivirus carrying the KFERQ-PS-Dendra reporter as before^12^. Cells were photoactivated by exposure to a 3.5 mA (current constant) light emitting diode (LED: Norlux, 405 nm) for 3 min and then plated in glass-bottom 96 well plates. At the desired times, cells were fixed with 4% PFA and imaged using high-content microscopy (Operetta system, Perkin Elmer). Images were quantified using the manufacturer’s software in a minimum of 1,200 cells in 9 independent fields per well. Although cell lines used in this study (NIH 3T3 and N2a) stably express the fluorescent reporter and the percentage of transduced cells is usually >85%, we set the program to identify number of cells by nuclei but to discount nuclei that did not have associated cytosolic green fluorescence. This was particularly important in the case of primary human cells where the efficiency of transduction was ~65%. To avoid confounding effects for drug-induced changes in cell volume or adherence to the plate that could bring a fraction of the cells per field outside of focus, we set a threshold to count only cells that have at least 1/10 of the average number of cells detected in the untreated wells for each cell type (3–4 puncta/cell in the case of NIH3T3 and N2a cells and 5 puncta/cell in primary human fibroblasts).

### Chemical synthesis of CMA activators

All chemical reagents and solvents were obtained from commercial sources (*Aldrich*, *Acros*, *Fisher*) and used without further purification unless otherwise noted. Chromatography was performed on a *Teledyne ISCO CombiFlash Rf 200i* using disposable silica cartridges (4, 12, and 24 g). Analytical thin layer chromatography (TLC) was performed on aluminum-backed *Silicycle* silica gel plates (250 µm film thickness, indicator F254). Compounds were visualized using a dual wavelength (254 and 365 nm) UV lamp, and/or staining with CAM (cerium ammonium molybdate) or KMnO_4_ stains. NMR spectra were recorded on *Bruker DRX 300* and *DRX 600* spectrometers. 1H and 13 C chemical shifts (d) are reported relative to tetramethyl silane (TMS, 0.00/0.00 ppm) as internal standard or to residual solvent (CDCl3: 7.26/77.16 ppm; dmso-d6: 2.50/39.52 ppm). Mass spectra (ESI-MS) were recorded on a *Shimadzu LCMS 2010EV* (direct injection unless otherwise noted). High resolution mass spectra (HRMS) were recorded on an *Orbitrap Velos* high resolution mass spectrometer at the Proteomics Facility of Albert Einstein College of Medicine.

Protocols for the synthesis of the CMA activators used in this study are described in a Supplementary Note.

### Expression and purification of RARα LBD

The ligand binding domain (LBD) of the human RARα (residues 176-421) was fused to a preceding His6-SUMO tag separated by a ubiquitin-like protease (ULP1) cleavage site and was expressed in *Escherichia coli* BL21(DE3) RIL cell strain. Cells were grown at 37 °C in LB medium supplemented with 50 µg/mL kanamycin until OD_600_ reached about 0.8. Expression of T7 polymerase was induced by addition of isopropyl-b-d-thiogalactoside (IPTG, #10724815001) to a final concentration of 0.8 mM and cells were incubated at 20 °C overnight. Cell cultures were harvested by centrifugation at 8,000 × g for 15 mins. The cell pellet from 1 liter of LB growth was resuspended in 50 mL lysis buffer (20 mM Tris-HCl pH 8, 500 mM NaCl, 25 mM imidazole) supplemented with one complete™, EDTA-free protease inhibitor tablet (Roche Applied Science, #11873580001). The suspension was lysed using a high pressure homogenizer and centrifuged at 35,000 × g at 4 °C for 45 minutes. The supernatant was filtered and loaded onto a prepared 5 ml Ni^2+^-affinity column (HisPur™ Ni-NTA resin, Thermo Scientific™, #A50588), preequilibrated with lysis buffer. The column was washed 3x with 15 mL lysis buffer. Bound proteins were eluted with lysis buffer containing 200 mM imidazole (Sigma, # I5513). Eluted protein was concentrated and buffer exchanged in FPLC buffer (20 mM Tris-HCl, pH 7.5, 150 mM NaCl, 1 mM DTT) using an Amicon^®^ Ultra-15 10 K centrifugal filter unit (Millipore Sigma, #UFC9010). The His6-SUMO tag was removed by ULP1 cleavage followed by subtractive affinity purification with HisPur™ Ni-NTA resin. The RARα LBD protein was further purified using a Superdex 200 Increase 10/300 GL gel filtration column (Fisher Scientific, #45002570) preequilibrated with FPLC buffer. Purified RARα fractions were pooled, 5 mM DTT was added, and protein was stored at 4 °C.

### Fluorescence polarization binding assays

The fluorescein-tagged peptide of N-CoR1, FITC-Ahx-RLITLADHICQIITQDFAR (FITC-N-CoR1) was provided by Genscript at purity > 95%. Fluorescence polarization assays (FPA) were performed using established procedures^36^. Direct binding isotherms were generated by incubating FITC-N-CoR1 (5 nM) with or without small molecules (10 μΜ) with serial dilutions of RARα LBD starting from 10 μΜ and diluted two-fold at each step. The buffer solution for assays was 20 mM Tris-HCl, pH 7.5, 150 mM NaCl, 1 mM EDTA, 5 mM DTT and 10% (v/v) glycerol. Fluorescence polarization was measured at 30 min on a F200 PRO microplate reader (TECAN) with the excitation wavelength set at 470 nm and emission measured at 530 nm. EC_50_ values were calculated by nonlinear regression analysis of competitive binding curves using GraphPad Prism software.

### Molecular docking and structural analysis

AR7, CA39 and CA77 structures were drawn in ChemDraw and converted to three-dimensional all-atom structures from sdf format using LigPrep (Schrödinger, LLC, Releases 2016-2020). For each ligand a maximum of 4 stereoisomers were generated, ionization states and tautomers were generated for pH 7 and pH 2 and geometries optimized and energy minimized before docking. The structure of the RARα-RXR hetero-dimer in complex with the small molecule antagonist BM614 (PDB ID: 1DKF), was used for docking. The RARα-RXR structure was prepared using MAESTRO protein preparations module (Schrödinger, LLC, Releases 2016-2020). The structure of the antagonist was removed from the RARα site, water molecules at a distance of more than 5 Å from heteroatoms were removed, all missing protons were generated, hydrogens were optimized for best hydrogen bonding network bonds and formal charges were assigned and structure was gently minimized by restrained energy minimization. The ligand-binding pocket was defined within 5 Å of the BMS614 pose, and receptor grid size and center was generated based on the position and the size of the BMS614. To account for receptor flexibility in docking, scaling of van der Waals’ radii of non-polar atoms with the absolute value of the partial atomic charge less than or equal to 0.25 for protein atoms was set to 1 and for ligand non-polar atoms with partial atomic charges less than or equal to 0.15 was set to 0.8. Docking was performed in ligand flexible mode using Glide (Schrödinger, LLC) using the extra precision (XP) mode. All three molecules were docked into the RARα site binding site. Similar procedures were performed for docking to RARα crystal structure bound to N-CoR1 peptide and BMS493 inverse agonist (PDB ID: 3KMZ) using extra precision (XP) and induced fit docking (IFD). Structures were analyzed using MAESTRO and PyMOL (Version 2.3, The PyMOL Molecular Graphics System, Schrödinger, LLC.).

### In vitro and in silico *ADME*

In vitro solubility in the following conditions A) HBSS/HEPES 10 mM/BSA 0.1% pH 6 B) HBSS/HEPES 10 mM/BSA 0.1% pH 7.4 C) TRIS/BSA 0.1% PBS (pH 7.4 and at indicated doses were measured by Nephelometry using the NEPHELOstar Galaxy apparatus (BMG Lab Technologies). In vitro stability in human, mouse and rat liver microsomes and permeability using a bidirectional permeability assay with CaCo-2 cells (pH 6.5/7.4) assays were performed with standard methodology and analyzed by LC-MS/MS. at SIMM-SERVIER joint Biopharmacy Laboratory. ADME properties for CA39 and CA77 were predicted by QikProp (Schrödinger, LLC, Releases 2016-2020) software.

### Pharmacokinetic analysis

ICR (CD-1) male mice were fasted at least three hours and water was available ad libitum before the study. Animals were housed in a controlled environment, target conditions: temperature 18 to 29 °C, relative humidity 30 to 70%. Temperature and relative humidity was monitored daily. An electronic time-controlled lighting system was used to provide a 12 h light/12 h dark cycle. 3 mice for each indicated time point were administered 30 mg/Kg CA39 or CA77 by oral gavage or 1 mg/Kg CA39 or CA77 by intravenous injection using 30% PEG-400, 65% D5W (5% dextrose in water), 5% Tween-80 vehicle. Mice were sacrificed, and brain samples were harvested at 0 h, 0.25 h, 0.5 h, 1 h, 2 h, 4 h, 8 h, 24 h, and analyzed for CA39 or CA77 levels using LC-MS/MS. Pharmacokinetics parameters were calculated using Phoenix WinNonlin 6.3. Experiments performed at SIMM-SERVIER joint Biopharmacy Laboratory.

### Retina processing and staining

#### Ex-vivo retinal cultures

After removal of the eyes, all non-relevant tissue was removed from the neuroretina, which was then placed, photoreceptors upside down, in Millicell support inserts (Millipore, #PICM0RG50) and maintained in DMEM with 1 μM insulin (SIGMA, #I2643) for 72 h at 37 °C in a 5% CO_2_ atmosphere. Where indicated, retinas were incubated with 40 μM CA77 for the indicated times. The retinas were then washed twice with phosphate-buffered saline (PBS), fixed overnight in 4% paraformaldehyde (w/v) in 0.1 M phosphate buffer (pH 7.4) and processed. Retinas were separated along dorsoventral axis and nasal half was used for whole-mounts and temporal half was used for 12 µm cryosections.

#### Staining of whole-mount retinas

Immunostainings were performed overnight at 4 °C using antibodies against visual (rod) arrestin and cone arrestin after initial permeabilization with 2% Triton X-100 for 1 h at RT and subsequent incubation with blocking solution (10% normal goat serum, 0.25% Triton X-100 in PBS). The retinas were then washed and incubated for 1 h with Alexa 568 or 647 (Invitrogen, #A-11004, #A32728), counterstained with DAPI (1 µg/mL, Sigma, # D9542), mounted in Fluoromount-G (Cultek, #100-01), and visualized by confocal microscopy in an SP5 confocal microscope (TCS SP5; Leica Microsystems).

#### Cryosections and immunofluorescence

12 μm cryosections and immunofluorescence in retinal sections^10^ after orienting retinas with a dorsal mark, so comparisons were performed taking into account the precise retinal area. Primary antibodies used in this study were visual (rod) arrestin, cone arrestin, Opsin R/G, transducin, GFAP, N-CoR1. Sections were visualized by confocal microscopy (TCS SP5; Leica Microsystems).

#### ONL thickness and outer segment length quantification

For ONL thickness quantification, DAPI images were taken at 40X in a fluorescence microscope Multidimensional system Leica AF6000 LX coupled to DMI600B microscope and Hamamatsu CCD 9100-O2 camera. At least two sections per animal were analyzed, preferably central sections. Eight images per retina equally distributed along the retina were acquired. Three measures were taken per image and ratio ONL/INL quantified with ImageJ tools. Leica LAS-X (3.7.4.23463) was used for image acquisition and ImageJ (v.2.1.0) was used for image processing. For OS length measures, fixed positions common to all pictures were considered and OS length was measured using ImageJ straight line tool.

#### Semithin sections

Eyes were enucleated, put in a plate with cold PBS and an incision was made under the microscope to let the fixative enter into the eye. Eyes were immersed in the fixative solution 2 h at RT while rotating and after taking out the lens, eyes were put on fresh fixative o/n at 4 °C in (2% PFA, 2% GA (Sigma, #G5882) in 0.1 M cacodilate buffer ph7.4 (CB, Sigma, #C4945). Next day we washed abundantly in 0.1 M CB (3 times for 15 minutes). Then, eyes were put under rotation in the solution 1% Osmium tetroxide, OsO_4,_ (Sigma, #75632, 1% Potassium ferrycianide (Sigma, #702587) diluted in distilled water for 2 h at 4 °C in dark. Next, they were washed abundantly with water (until no yellow color in detectable). Then, we dehydrate the samples following 50%, 70%, 80%, 90%, 95%, and twice 100% for 10 min each in shaker. Finally, we underwent the inclusion process by incubating 3 times in propylene oxyde (Sigma, #540048) for 10 min. To infiltrate the tissue with the resin Epon (EMS:Embed 812 #14120), we use propylene oxide:Epon resin 3:1 (1 h), propylene oxide:Epon resin 1:1 (overnight), propylene oxide:Epon resin 1:3 (1 h) and pure Epon resin 2 times for 1 h, all in the shaker at RT. Finally, we included the eyes oriented in Epon in the mold in the oven at 60 °C for 12 h. For visualization, 0.5 µm-semithin sections were stained with toluidine blue (Electron Microscopy Sciences, #22050).

### ERG recordings

Mice were dark-adapted overnight, and subsequent manipulations were performed in dim red light. Mice were anesthetized with intraperitoneal injections of ketamine (95 mg/kg) (Ketolar; Pfizer, #631028.1) and xylazine (5 mg/kg) (AnaSed, NADA # 139-236) solution and maintained on a heating pad at 37 °C. Pupils were dilated with a drop of 1% tropicamide (Colircusi Tropicamida; Alcon Cusi, #653486). To optimize electrical recording, a topical drop (2% Methocel; Omnivision GmbH) was instilled on each eye immediately before situating the corneal electrode. Flash-induced ERG responses were recorded from the right eye in response to light stimuli produced with a Ganzfeld stimulator. Light intensity was measured with a photometer at the level of the eye (Mavo Monitor USB; Nürenberg). Four to 64 consecutive stimuli were averaged with an interval between light flashes in scotopic conditions of 10 s for dim flashes and of up to 60 s for the highest intensity. Under photopic conditions, the interval between light flashes was fixed at 1 s. ERG signals were amplified and band-filtered between 0.3 and 1000 Hz with an amplifier (CP511 AC amplifier; Grass Instruments). Electrical signals were digitized at 20 kHz with a power laboratory data acquisition board (AD Instruments). Bipolar recording was performed between an electrode fixed on a corneal lens (Burian-Allen electrode; Hansen Ophthalmic Development Laboratory) and a reference electrode located in the mouth, with a ground electrode located in the tail. Under dark adaptation, scotopic threshold responses (STR) were recorded to light flashes of −4 log cd·s·m^−2^; rod responses (b-scot) were recorded to light flashes of −2 log cd·s·m^−2^s and mixed (b-mixed) responses were recorded in response to light flashes of 1.5 log cd·s·m^−2^. Oscillatory potential (OP) was isolated using white flashes of 1.5 log cd·s·m^−2^ in a recording frequency range of 100 to 10,000 Hz. Under light adaptation, cone-mediated responses to light flashes of 2 log cd·s·m^−2^ on a rod-saturating background of 30 cd·m^−2^ were recorded (b-phot). Wave amplitudes of the STR, rod responses (b-rod), mixed responses (a-mixed and b-mixed) and OP were measured under dark adaptation and wave amplitudes of the cone responses (b-phot) and flicker response (flicker) were measured under light adaptation. All measurements were performed off line by an observer masked to the experimental condition of the animal.

### mRNA quantification

#### mRNA-qPCR

RNA was extracted from individual retinas or tissue sections (brain, liver). Total RNA from retinas was extracted using TRIzol Reagent (Invitrogen, # 15596-018), and reverse transcription was performed using the High-Capacity cDNA Reverse Transcription Kit (Applied Biosystems, # 4374966) according to the manufacturer’s instructions. Quantitative real-time PCR was performed in a Light Cycler 480 Instrument (Roche) with Taqman LightCycler 480 probes Master Mix (Roche, #4887301001) using Taqman assays (Life Technologies). For cells in culture, RNA was extracted using RNeasy Plus Mini Kit (Qiagen, #74034), and reverse transcription was performed using Superscript III (Invitrogen, #18080051). Quantitative RT-PCR analysis were performed using Power SYBR Green PCR Master Mix (Applied Biosystems, #4368708) on a StepOne Plus Real-Time PCR System (Applied Biosystems). Mm01184405_m1 probe (Thermofisher) was used for rhodopsin and the sequence of all the other probes used for qPCR is included in Supplementary Table 2.

#### Microarray

Total RNA of the treated cells was extracted using TRIzol (Invitrogen) and purified with RNeasy chromatography (Qiagen). Cy3-labeled RNA (0.6 μg) from each condition was hybridized to Agilent Mouse 8x60K. Data were processed using the oligo package and normalized using Robust Multiarray Average (RMA) method. Gene set was filtered to remove genes without Entrez or GO annotation (21912 genes out of 55682) and genes with an IQR >0.5. Pathway analysis was performed using the STRING database (https://string700db.org/).

### Co-immunoprecipitation and immunoblot

#### Co-immunoprecipitation

Cells were lysed in 25 mM Tris, pH 7.2, 150 mM NaCl, 5 mM MgCl_2_, 0.5% NP-40, 1 mM DTT, 5% glycerol and protease inhibitors for 15 min on ice and then centrifuged for 15 min at 16,000 g. Supernatant was precleared with Protein A/G sepharose and then incubated with the primary antibody overnight at 4 °C with continuous rocking. Protein A/G sepharose was added to the tubes and after incubation in the same conditions for 1 h, samples were spun and supernatant (FT, flow through) and beads (IP, immunoprecipitate) were subjected to SDS-PAGE and immunoblot.

#### Immunoblot

Cells were lysed in RIPA and neuroretinas in a buffer containing 50 mM Tris-HCl (pH 6.8), 10% glycerol (v/v), 2% SDS (w/v), 10 mM DTT, and 0.005% bromophenol blue. Protein concentration was determined using the Lowry method with bovine serum albumin as standard^37^. Fifty micrograms of protein (for cell lysates) or 15 micrograms of protein for neuroretinas were resolved on AnyKD SDS-PAGE gel (BioRad, #5678124). The proteins were then transferred to PVDF membranes (Bio-Rad, #1704273), which were blocked for 1 h in PBS-Tween 20 (0.05% (v/v)) containing 5% non-fat milk and then probed with primary and secondary antibodies. Antigen signals were detected using the appropriate horseradish peroxidase-labelled secondary antibodies (Pierce) and were visualized with the SuperSignal West Pico chemiluminescent substrate (Pierce, #34580). Densitometric analysis was performed with Quantity One software (v.4.6.8., Bio-Rad). Full scan uncropped and unprocessed blots are included in Supplementary Fig. 10.

### Histopathology of peripheral organs and blood cell count

Liver, lung and kidneys from CA-treated animals were dissected and fixed in 1% PFA overnight and paraffin-embedded. Tissues were sectioned, stained with hematoxylin and eosin (H&E), and analyzed by an expert pathologist, blind to the treatment groups, to score for the possible presence of toxicity in these organs. Scoring 1-6 was used assigning 6 to those observations clinically relevant. Individual scoring per parameter and per organ and average scoring per organ were performed. Blood cell count in the groups of mice administered vehicle or CA was analyzed in tail blood drawn monthly and at the moment of tissue dissection using an Oxford Science Forcyte Blood Analysis Unit.

### KFERQ-Dendra mice tissues imaging

All mice were perfused with PBS. Livers were post-fixed overnight at 4 °C in picric acid fixation buffer (2% formaldehyde, 0.2% picric acid in PBS, pH7.0) and then washed with 70% ethanol, followed by two washes in PBS. Livers were immersed in 30% sucrose and then embedded in OCT for sectioning in a cryostat (Leica CM3050 S). After airdrying for 30 min, sections were stored at −20 °C until use. Brains were post-fixed for 12 h in 4% paraformaldehyde, cryoprotected in PBS containing 20% sucrose before being frozen by immersion in a cold isopentane bath (−50 °C), and stored immediately at −80 °C until sectioning. Brains were also sectioned in a cryostat at −20 °C in coronal 40 μm-thick free-floating sections. Immunostainings were performed after permeabilization of the sections with 0.3% Triton X-100 in PBS for 30 minutes at RT followed by blocking (10% normal goat serum, 2% Triton X-100 in PBS) and subsequent incubation with the primary and appropriate anti-species secondary antibody. Cell nuclei were stained using Hoechst (Life Technologies, 33342) at 1:5000 for 30 s (in retina) or 10 min (in brain sections) prior to mounting. Images were acquired with a Leica confocal TCS-SP8 (Leica Microsystem) using an HCX Plan Apo CS ×63.0 1.40 NA oil objective and the Leica Application Suite X (LAS X). Puncta was quantified using ImageJ Software (NIH)^38^ after thresholding. Where indicated, the invert function was used in a single channel of interest to create an inverted mask for better visualization of the cytosolic region. Image analyses were performed blind to the intervention or genotype.

### Calculation of CMA activation index

CMA index^14^ was calculated using data set from^23–25^. Briefly, each element of the CMA network^13^ has attributed a weight. As LAMP-2A is the rate-limiting component of CMA, it was given a weight of 2. Every other element received a weight of 1. Then, every element was attributed direction score that is +1 or −1 based on the known effect of a given element on CMA activity. The score was then calculated as the weighted/ directed average of expression counts of every element of the CMA network.

### Statistical analysis and sample size determination

All numerical results are reported as mean ± s.e.m. and represent data from a minimum of three independent experiments unless otherwise stated. Statistical significance of difference between groups was determined in instances of single comparisons by the two-tailed unpaired Student’s *t*-test of the means. In instances of multiple means comparisons, we used one-way analysis of variance (ANOVA) followed by the Bonferroni post-hoc test to determine statistical significance. In the studies of resistance to oxidative stress in contralateral retinas treated with vehicle or CA, data transformation was applied as indicated in the legend of the figure. Statistical analysis was performed in all of the assays, and significant differences are noted in the graphical representations. If assumptions of normality and homoscedasticity were not met, we applied non-parametric tests. In studies with data normalized over control we used one sample *t* test with hypothesized control mean of 1. For all tests the significance level was p < 0.05 (2-tailed). The number of animals used per experiment was calculated through power analysis with the G*Power software (v.3.1.9.7, RRID: SCR_013726) based on previous results for a significance of 0.05 and a statistical power of 0.8. Animals were randomly attributed to each treatment group using the SELECT BETWEEN RANGE function in Microsoft Excel. No mouse was excluded from the analysis unless there was technical reason, or the mouse was determined to be in very poor health by the veterinarian. For the studies involving cells in culture treatment groups were attributed randomly between wells and plates to account for well or tube positioning effects. We determine number of experimental repetitions to account for technical variability and changes in culture conditions based on our previous studies using those systems. Every experiment was performed in at least 3 independent replicates. Experiments in cells in culture were performed on different days to confirm the reproducibility of the procedures. All independent replications were successful. For molecular docking studies, sample size was chosen based on previous experience with similar studies. Outliers were determined by the ROUT method (Q = 1%). Investigators were blinded to the treatment during data collection and analysis for both in vivo and in vitro experiments and unblinding was done when the analysis was completed for plotting and image composition of the figures. Basic data handling was done in Microsoft Excel 365 (v.16.57 and v.2101). Data analysis was performed with Prism software (v9 - Graph Pad Software Inc). Image analysis and quantification were performed using ImageJ (v.2.1.0). Immunoblot quantification with Quantity One software (v..6.8., Bio-Rad). Structural and small molecule data were analyzed with modules GLIDE, EPIK, LIGPREP, QIKPROP, MAESTRO of Schrodinger software suite (Releases 2016-2021).

### Reporting summary

Further information on research design is available in the Nature Research Reporting Summary linked to this article.

## Supplementary information

Supplementary Informaton

Reporting Summary

## Data Availability

There are no restrictions on data availability in this manuscript. The data in main and supplementary figures generated in this study are provided in the Supplementary Information/Source Data file as an Excel worksheet organized by figures and inside each figure sheet by panels and it includes raw data and a full statistic report. Transcriptomic data has been deposited in the Gene Expression Omnibus (GEO) database under accession code GSE194291 and GSE194292. The structural data used in this study are available in the PDB protein data bank under accession codes 1DKF and 3KMZ. Source data are provided with this paper.

## Source data

Source Data
