# Supplementary material for: Targeting retinoic acid receptor alpha-corepressor interaction activates chaperone-mediated autophagy and protects against retinal degeneration
Source: Nat Commun. 2022 Jul 21;13:4220. doi: 10.1038/s41467-022-31869-1 (PMC9304322; doi:10.1038/s41467-022-31869-1)
Supplement: Supplementary file 1 — Supplementary Informaton [file 41467_2022_31869_MOESM1_ESM.pdf]

Targeting retinoic acid receptor alpha-corepressor interaction activates chaperone-mediated autophagy and protects against retinal degeneration

Raquel Gomez-Sintes Qisheng Xin, Juan Ignacio Jiménez-Loygorri, Mericka McCabe, Antonio Diaz, Thomas P. Garner, Xiomaris M. Cotto-Rios, Yang Wu, Shuxian Dong, Cara A Reynolds, Bindi Patel, Pedro de la Villa, Fernando Macian, Patricia Boya*, Evripidis Gavathiotis*, Ana Maria Cuervo*

Supplementary Figures 1 – 10

Supplementary Note (compound synthesis)

Supplementary Tables 1-2

Source Data

- Supplementary Data file (raw data) in separate Excel File

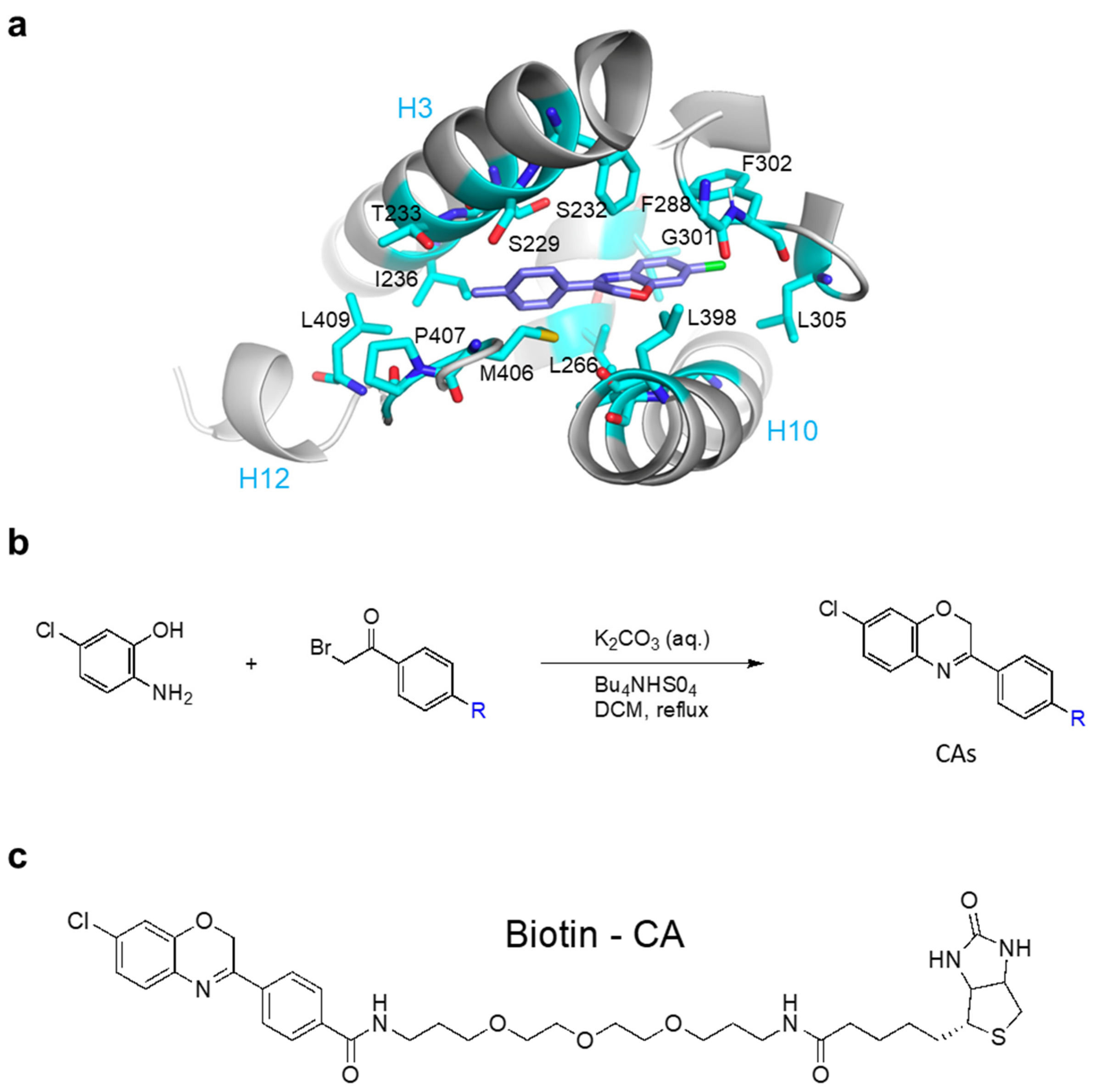

Supplementary Fig. 1. **Structure-based design and chemical synthesis of CMA activators (CA compounds). a.** A close view of the binding pose of AR7 (purple sticks) in the binding pocket of inactive RARα in ribbon (grey) highlighting RARα interacting residues in sticks (cyan). AR7 occupies a hydrophobic pocket present in the inactive RARα formed by helices h3, h10 and h12. **b.** New CA compounds were designed and synthesized according to a synthetic approach varying R groups aiming to increase hydrophobic interactions or polar interactions within the RAR pocket residues. **c.** Conjugation of biotin to a CA compound to generate Biotin-CA compound that was used in the pulldown experiments.

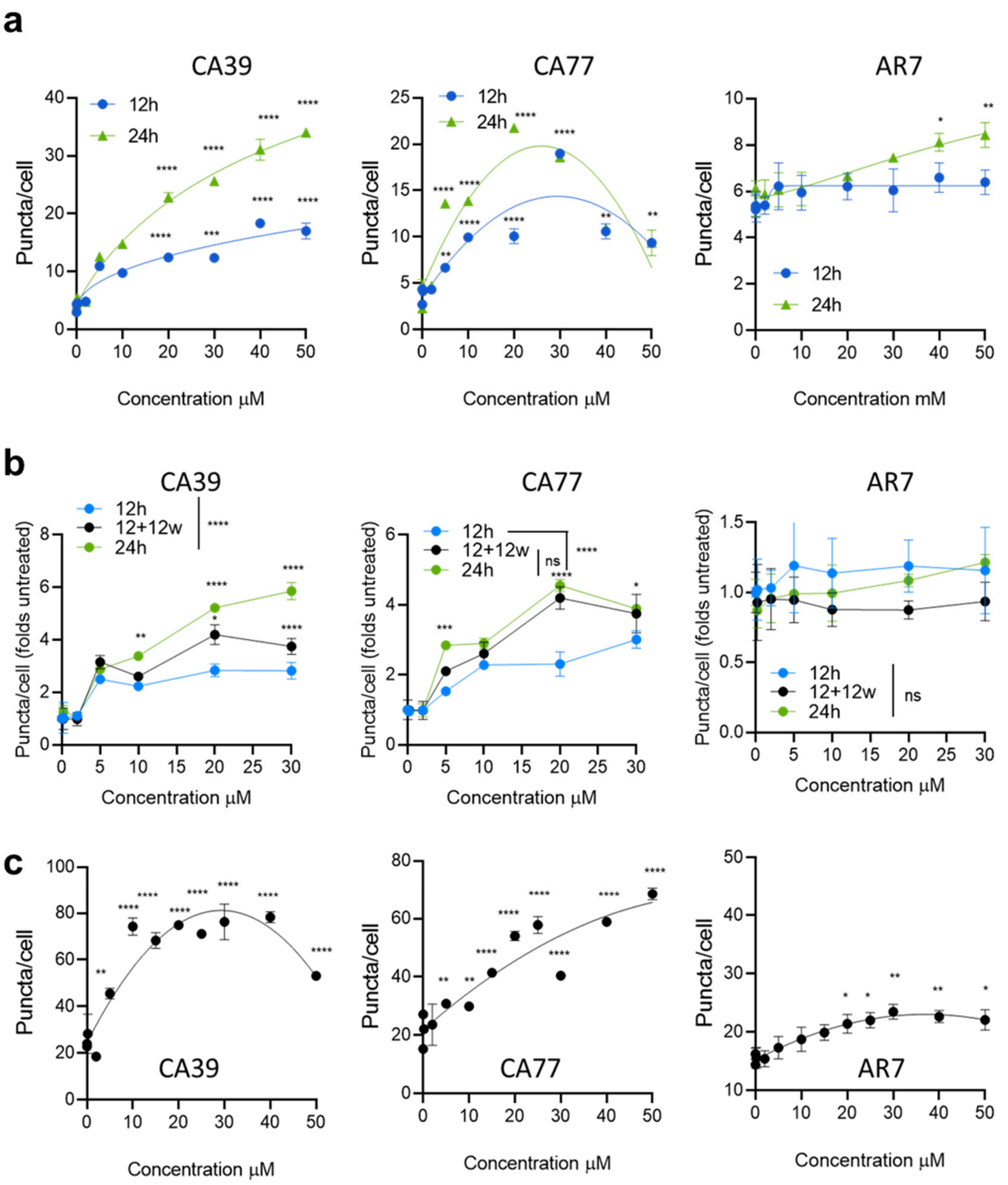

Supplementary Fig. 2. **Activation of CMA by CA39, CA77 and AR7 in different cell types. a, b**. CMA activity in neuroblastoma cell line N2a expressing the KFERQ-PS-Dendra reporter upon addition of increasing concentrations of CA39 (left), CA77 (center) or AR7 (right) for the indicated times (**a**) or 12 h after washing (w) them out from the media (**b**). n = 3 independent experiments (>1,500 cells counted). **c.** CMA activity in primary human fibroblast expressing the KFERQ-PS-Dendra reporter upon addition of increasing concentrations of CA39 (left), CA77 (center) or AR7 (right) for 24 h. n =3 independent experiments (>850 cells counted). All values are mean+s.e.m. One-way ANOVA (**a, c**) or two-way ANOVA (**b**) followed by Bonferroni's multiple comparisons post-hoc test were used. Significant differences with untreated samples are indicated in a, c and among the different incubation protocols in **b**. **p<0.01, ***p<0.001, ****p<0.0001. ns: not significant. Source data and exact p values are provided as a Source Data file.

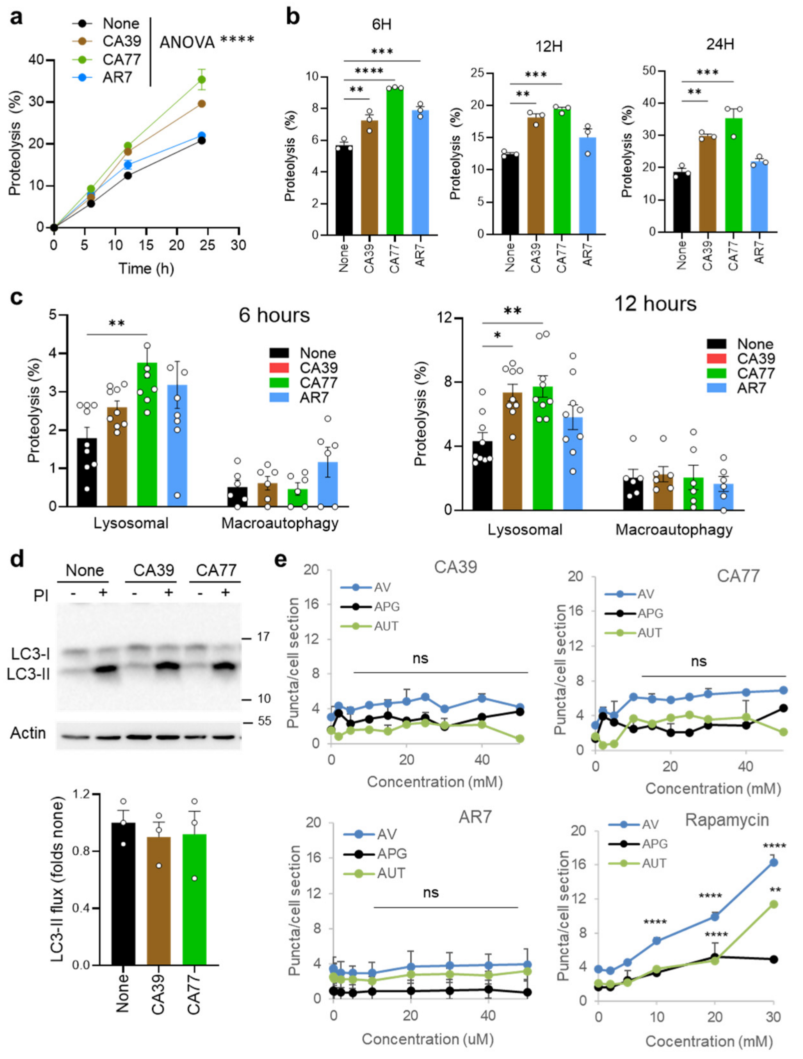

Supplementary Fig. 3. **Lack of effect of CA39 and CA77 on macroautophagy activity**. **a, b.** Proteolysis of long half-life intracellular proteins in NIH3T3 without additions (None) or cultured in the presence of 10 μM CA39, CA77 or AR7 for the indicated times. Proteolysis rates at the indicated times are shown in b. n = 3 independent experiments with triplicate wells. **c**. Proteolysis of long half-life intracellular proteins in cells incubated with NH_4Cl/Leupeptin to calculate the percentage of lysosomal-dependent proteolysis or with MRT o clacualte macroautophagy-dependent proteolysis. n = 3 independent experiments with triplicate wells. **d.** Immunoblot for LC3 in NIH3T3 incubated with 20 μM CA39 or CA77 for 16h in presence or not of lysosomal protease inhibitors (PI) added in the last 6h of the experiment. Representative immunoblot (left) and quantification of the rate of degradation of LC3-II (flux) relative to that in untreated (None) cells (right). n = 4 independent experiments. **e.** Quantification of macroautophagy activity in NIH3T3 cells stably expressing the mCherry-GFP-LC3 reporter and treated with increasing concentrations of CA39, CA77, AR7 or the macroautophagy activator rapamycin for 16h. Quantification of the amount of $mCherry^+$ puncta (autophagic vacuoles, AV), $mCherry^+GFP^+$ puncta (autophagosomes, APG) and $mCherry^+GFP^-$ puncta (autolysosomes, AUT). n = 3 different experiments (>1,100 cells counted). All values are mean+s.e.m. One-way ANOVA (b,d) or two-way ANOVA (a,c,e) followed by Bonferroni's multiple comparisons post-hoc test (a) were used. A. *p<0.05, **p<0.01 ***p<0.001 and ****p<0.0001. ns: not significant. Uncropped blots are in Supplementary Fig. 1. Source data and exact p values are provided as a Source Data file.

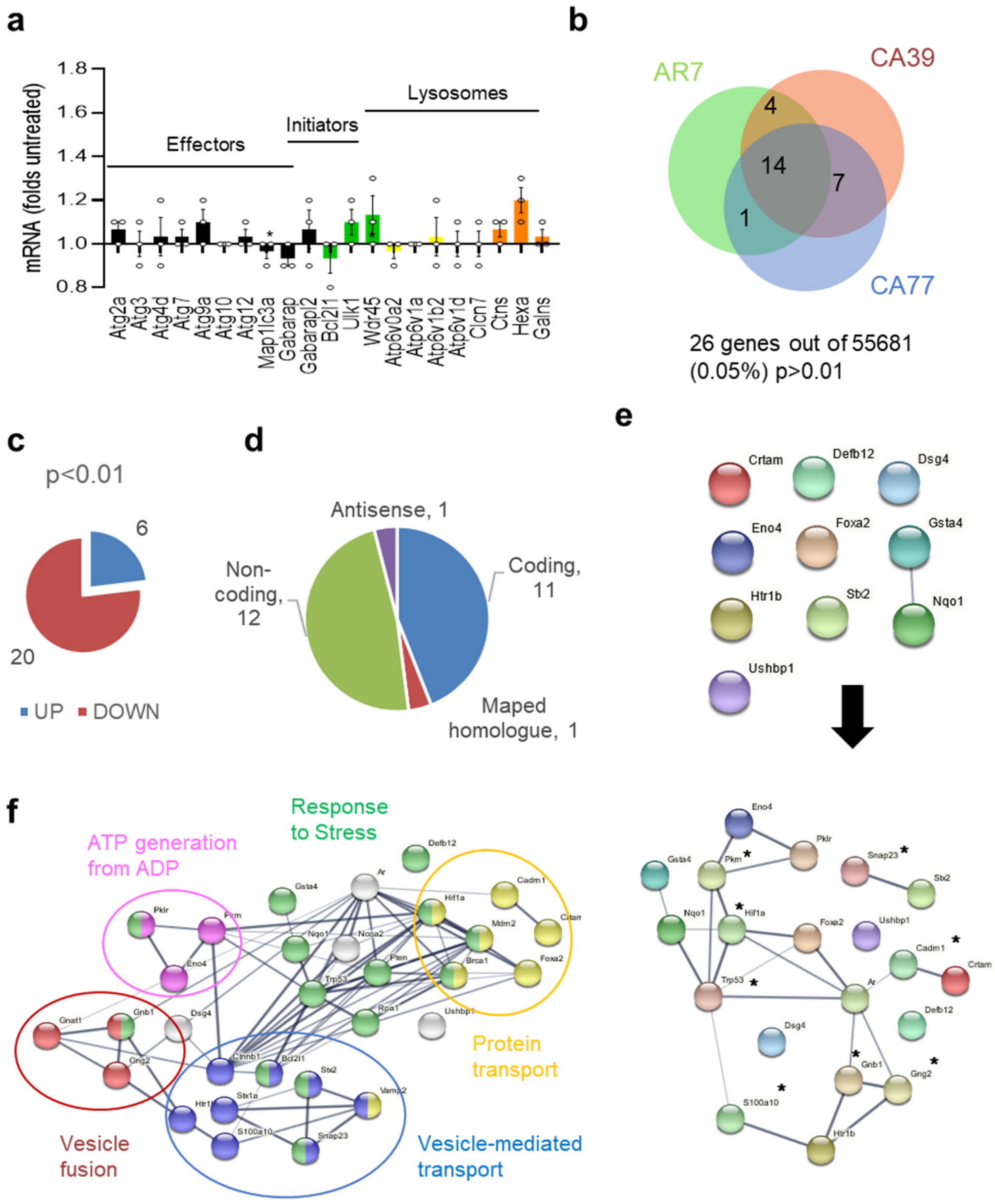

Supplementary Fig. 4. **Transcriptional changes induced by CA compounds. a.** AR7 induced changes in the expression of the indicated components of the CLEAR network (macroautophagy and lysosomal examples shown). Values are expressed relative to untreated cells and are mean+s.e.m. n = 3 different experiments. **b.** Venn diagram of the additional genes (beside those in the CMA network) showing significant ($p<0.01$) changes in expression in cells treated with the indicated compounds. **c.** Fraction of genes significantly ($p<0.01$) upregulated or downregulated upon treatment with AR7, CA39 and CA77. **d.** Types of transcripts with modified expression upon treatment with AR7, CA39 and CA77. **e.** Proteins coded by the genes significantly changing upon treatment with AR7, CA39 and CA77. Below shows their clustering upon gene set enrichment and node expansion analysis (using STRING database). *: proteins added through node expansion. **f.** Functional groups of the nodes assigned to the 11 proteins with expression changed upon treatment with AR7, CA39 and CA77. One sample t and Wilcoxon test was used in **a** and unpaired two tails t-test (**c**)**.** *$p<0.05$. Source data and exact p values are provided as a Source Data file.

a

Product Name	Batch Name	Solubility		Metabolic Stability		Permeability		Comments
		Media Description	Minimal Soluble Concentration (μM)	Species	Calculated % MF	Fab%	Efflux Ratio	
CA39	20140811	A	2.0	Human	100	0.00	0.706	Low solubility; High metabolic stability (in human); Very low permeability.
CA39	20140811	B	2.0	Mouse	47			
CA39	20140811	C	10.0	Rat	70			
CA77	20140811	A	20.0	Human	23	92.0	2.452	Intermediate solubility; Intermediate metabolic stability (in human); High permeability.
CA77	20140811	B	10.0	Mouse	33			
CA77	20140811	C	20.0	Rat	40			

b

CA39

```
Primary Metabolites & Reactive FGs:
> Metabolism likely: benzylic-like H -> alcohol
Principal Descriptors:                      (Range 95% of Drugs)
  Solute    Molecular Weight     =  319.790  ( 130.0 / 725.0)
  Solute    Dipole Moment (D)    =    3.548  (   1.0 /  12.5)
  Solute    Total      SASA      =  580.127  ( 300.0 /1000.0)
  Solute    Hydrophobic  SASA    =   42.169  (   0.0 / 750.0)
  Solute    Hydrophilic  SASA    =    8.276  (   7.0 / 330.0)
  Solute    Carbon Pi    SASA    =  458.081  (   0.0 / 450.0)*
  Solute    Weakly Polar SASA    =   71.602  (   0.0 / 175.0)
  Solute    Molecular Volume (A^3)=  996.627 ( 500.0 /2000.0)
  Solute    vdW Polar SA (PSA)   =   18.774  (   7.0 / 200.0)
  Solute    No. of Rotatable Bonds=   1.000  (   0.0 /  15.0)
  Solute as Donor -    Hydrogen Bonds =   0.000  (  0.0 /   6.0)
  Solute as Acceptor - Hydrogen Bonds =   1.750  (  2.0 /  20.0)*
  Solute Globularity   (Sphere = 1)  =   0.832  ( 0.75 /  0.95)
  Solute Ionization Potential (eV)   =   8.791  (  7.9 /  10.5)
  Solute Electron Affinity    (eV)   =   1.030  ( -0.9 /   1.7)
Predictions for Properties:
 QP Polarizability (Angstroms^3)     =  37.464M (  13.0 /  70.0)
 QP log P  for   hexadecane/gas      =  11.215M (   4.0 /  18.0)
 QP log P  for   octanol/gas         =  13.599M (   8.0 /  35.0)
 QP log P  for   water/gas           =   5.816M (   4.0 /  45.0)
 QP log P  for   octanol/water       =   5.755  (  -2.0 /   6.5)
 QP log S  for   aqueous solubility  =  -6.444  (  -6.5 /   0.5)
 QP log S - conformation independent =  -6.306  (  -6.5 /   0.5)
 QP log K hsa Serum Protein Binding  =   1.001  (  -1.5 /   1.5)
 QP log BB for    brain/blood        =   0.599  (  -3.0 /   1.2)
 No. of Primary Metabolites          =      1   (   1.0 /   8.0)
 Predicted CNS Activity (-- to ++)   =     ++
 HERG K+ Channel Blockage: log IC50  =  -6.439  (concern below -5)
 Apparent Caco-2 Permeability (nm/sec) =  8268  (<25 poor, >500 great)
 Apparent MDCK  Permeability (nm/sec)  = 10000  (<25 poor, >500 great)
 QP log Kp for skin permeability     =   0.038  (Kp in cm/hr)
 Jm, max transdermal transport rate  =   0.126  (micrograms/cm^2-hr)
 Lipinski Rule of 5 Violations       =      1   (maximum is 4)
 Jorgensen Rule of 3 Violations      =      1   (maximum is 3)
 % Human Oral Absorption in GI (+-20%) =  100   (<25% is poor)
 Qual. Model for Human Oral Absorption =  low   (>80% is high)
```

CA77

```
Primary Metabolites & Reactive FGs:
> Metabolism likely: benzylic-like H -> alcohol
Principal Descriptors:                      (Range 95% of Drugs)
  Solute    Molecular Weight     =  300.744  ( 130.0 / 725.0)
  Solute    Dipole Moment (D)    =    6.762  (   1.0 /  12.5)
  Solute    Total      SASA      =  551.791  ( 300.0 /1000.0)
  Solute    Hydrophobic  SASA    =  136.573  (   0.0 / 750.0)
  Solute    Hydrophilic  SASA    =   65.923  (   7.0 / 330.0)
  Solute    Carbon Pi    SASA    =  277.694  (   0.0 / 450.0)
  Solute    Weakly Polar SASA    =   71.602  (   0.0 / 175.0)
  Solute    Molecular Volume (A^3)=  926.680 ( 500.0 /2000.0)
  Solute    vdW Polar SA (PSA)   =   56.391  (   7.0 / 200.0)
  Solute    No. of Rotatable Bonds=   1.000  (   0.0 /  15.0)
  Solute as Donor -    Hydrogen Bonds =   1.000  (  0.0 /   6.0)
  Solute as Acceptor - Hydrogen Bonds =   4.250  (  2.0 /  20.0)
  Solute Globularity   (Sphere = 1)  =   0.833  ( 0.75 /  0.95)
  Solute Ionization Potential (eV)   =   8.755  (  7.9 /  10.5)
  Solute Electron Affinity    (eV)   =   0.980  ( -0.9 /   1.7)
Predictions for Properties:
 QP Polarizability (Angstroms^3)     =  32.942M (  13.0 /  70.0)
 QP log P  for   hexadecane/gas      =   9.883M (   4.0 /  18.0)
 QP log P  for   octanol/gas         =  15.306M (   8.0 /  35.0)
 QP log P  for   water/gas           =   8.840M (   4.0 /  45.0)
 QP log P  for   octanol/water       =   3.455  (  -2.0 /   6.5)
 QP log S  for   aqueous solubility  =  -5.043  (  -6.5 /   0.5)
 QP log S - conformation independent =  -4.523  (  -6.5 /   0.5)
 QP log K hsa Serum Protein Binding  =   0.278  (  -1.5 /   1.5)
 QP log BB for    brain/blood        =   0.046  (  -3.0 /   1.2)
 No. of Primary Metabolites          =      1   (   1.0 /   8.0)
 Predicted CNS Activity (-- to ++)   =      +
 HERG K+ Channel Blockage: log IC50  =  -5.558  (concern below -5)
 Apparent Caco-2 Permeability (nm/sec) =  2348  (<25 poor, >500 great)
 Apparent MDCK  Permeability (nm/sec)  =  3071  (<25 poor, >500 great)
 QP log Kp for skin permeability     =  -1.660  (Kp in cm/hr)
 Jm, max transdermal transport rate  =   0.071  (micrograms/cm^2-hr)
 Lipinski Rule of 5 Violations       =      0   (maximum is 4)
 Jorgensen Rule of 3 Violations      =      0   (maximum is 3)
 % Human Oral Absorption in GI (+-20%) =  100   (<25% is poor)
 Qual. Model for Human Oral Absorption =  HIGH  (>80% is high)
```

Supplementary Fig. 5. ***In vitro* and *in silico* ADME of CA compounds. a.** In vitro solubility, metabolic stability (in liver microsomes from the indicated species) and permeability evaluation of CA39 and CA77, see Methods for experimental conditions. Comments on properties were added by an observer blinded to the nature of the compounds and the study. **b.** *In silico* QikProp (Schrödinger, LLC) analysis and ADME predictions for CA39 (left) and CA77 (right). MF: microsomal fraction; Fab: absorption; FG: functional groups; SASA: solvent-accessible surface area; PSA: polar surface area; eV: electron volts; QP: QikProp (Schrödinger, LLC QikProp derived value); MDCK: Madin-Darby Canine Kidney (Cells); Kp: skin permeability coefficient; Jm: predicted maximum transdermal transport rate; vdW: van der Waals; ADME: Absorption, distribution, metabolism, and excretion.

a

PK CA39 plasma

	$T_{1/2}$ (h)	T_{max} (h)	C_{max} (ng/mL)	AUC_{last} (h*ng/mL)	AUC_{INF_obs} (h*ng/mL)	$CL_{\_obs}$ (mL/min/kg)	MRT_{INF_obs} (h)	$Vss_{\_obs}$ (mL/kg)
p.o.	7.51	0.50	109	980	1103	-	10.9	-
i.v.	8.20	-	-	808	913	18.3	10.7	11722

PK CA77 plasma

	$T_{1/2}$ (h)	T_{max} (h)	C_{max} (ng/mL)	AUC_{last} (h*ng/mL)	AUC_{INF_obs} (h*ng/mL)	$CL_{\_obs}$ (mL/min/kg)	MRT_{INF_obs} (h)	$Vss_{\_obs}$ (mL/kg)
p.o.	2.19	2.00	278	1317	1458	343	3.97	-
i.v.	0.635	0.0830	36.2	35.3	35.8	465	0.952	26548

b

PK CA39 brain

Brain	$T_{1/2}$ (h)	T_{max} (h)	C_{max} (ng/g)	AUC_{last} (h*ng/g)	AUC_{INF_obs} (h*ng/g)	MRT_{INF_obs} (h)	$AUC_{b/p}$
p.o.	9.99	2.0	775	10214	12571	15.1	10.3
i.v.	8.10	-	-	8617	10007	13.1	10.1

PK CA77 brain

Brain	$T_{1/2}$ (h)	T_{max} (h)	C_{max} (ng/g)	AUC_{last} (h*ng/g)	AUC_{INF_obs} (h*ng/g)	MRT_{INF_obs} (h)	$AUC_{b/p}$
p.o.	1.77	1.00	30964	165622	165648	3.94	126
i.v.	4.20	0.250	3654	5499	5518	1.66	156

c

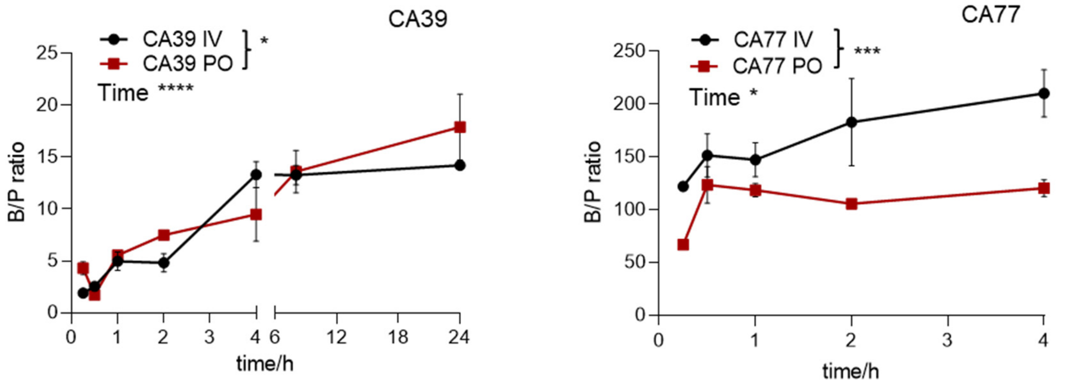

Supplementary Fig. 6. **CA compounds have favorable blood brain barrier penetration and pharmacokinetics. a, b.** Pharmacokinetics (PK) parameters of CA39 and CA77 in plasma (**a**) and brain (**b**) after p.o. (oral, 30 mg/kg bw) and i.v. (intravenous, 1 mg/kg bw) administration in mice. **c.** Brain to plasma (B/P) ratio of CA39 and CA77 at the indicated times after administration by i.v. or p.o. as in **a**. n = 3 mice per time point. All values are mean+s.e.m. Two-way ANOVA followed by Sidak's multiple comparisons post-hoc test was used in **c**. * p<0.05, ***p<0.001 and ****p<0.0001. ns: not significant. Source data and exact p values are provided as a Source Data file.

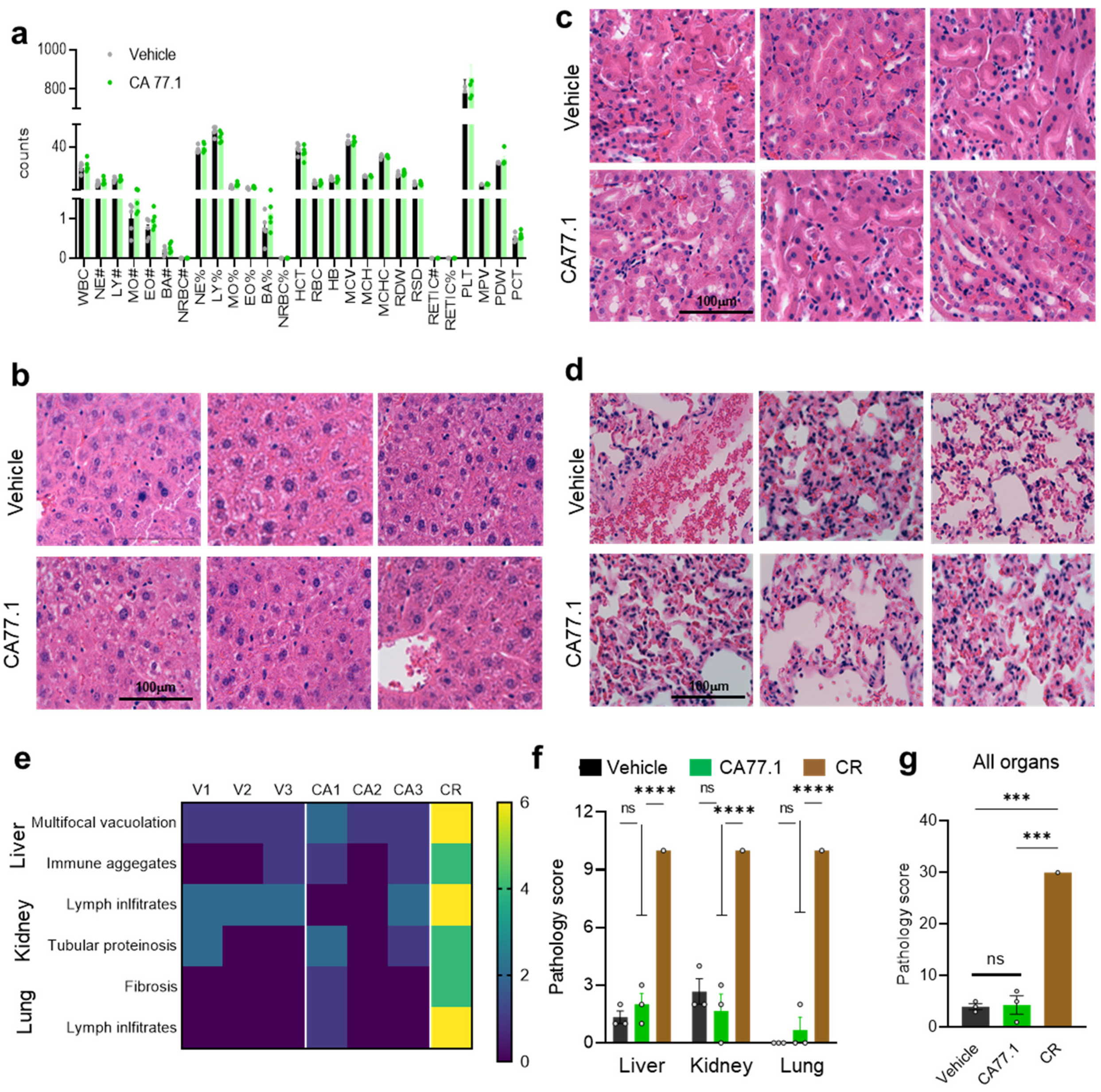

Supplementary Fig. 7. **Toxicity assessment of the CA compounds *in vivo*. a.** Mouse blood count at the end of a 5 month daily oral administration of vehicle (Veh) or a CA77 structure analogue (CA77.1). Values are mean+s.e.m. from n = 6 mice in each group. **b-d.** Representative images of sections of H&E stained liver (b), kidney (c) and lung (d) sections from the same mice. n = 5 mice All values are mean+s.e.m. Two-way ANOVA followed by Bonferroni's multiple comparisons post-hoc test and unpaired t test were both applied on A and no statistical differences were noted between vehicle and CA treated mice. **e-g.** Pathology scoring of the identified features in the three organs. Clinical relevant (CR) values are depicted as reference. Heat map of the individual features per organ (**e**), average scoring of all the features in each organ (**f**) or average of histological features per animal (**g**) are shown. n = 3 mice per group. All values are mean+s.e.m. Two-way ANOVA (**f**) or one-way ANOVA (**g**) followed by Tukeys' multiple comparisons post-hoc test were used. ***p<0.001 and ****p<0.0001. ns: not significant. Source data and exact p values are provided as a Source Data file.

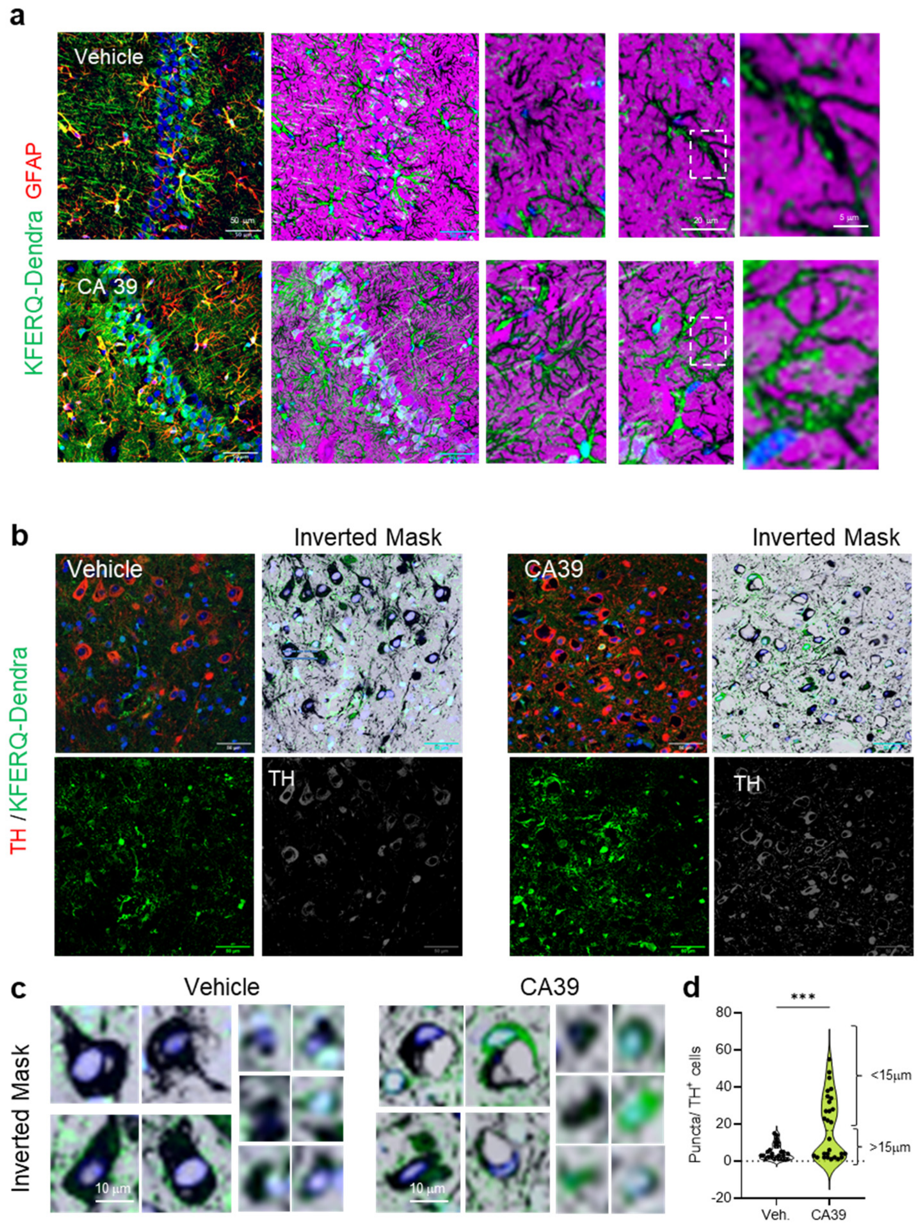

Supplementary Fig. 8. **Effect of CMA compounds on CMA activity of different brain cells *in vivo*. a.** Representative images of hippocampus (CA1 region) from the KFERQ-Dendra mice i.p. injected with vehicle or CA39 daily (30 mg/kg bw) for three consecutive days co-stained with GFAP. Merged channels (left) and inverted mask for the GFAP channel (right) to better appreciate Dendra$^+$ puncta are shown. Insets: single cells at boxed area at higher magnification. **b.** Representative images of midbrain substantia nigra regions from the same mice as in **a** co-stained with TH. Merged and individual channels are shown. Top right shows inverted mask for the TH channel to better appreciate Dendra$^+$ puncta. **c.** Higher magnification of examples of large (left) and small (right) TH$^+$ cells from the inverted mask images in **a. d.** Violin plot of the quantification of the number of Dendra$^+$ puncta in individual TH$^+$ cells from images as in **a.** Average sizes for neurons in the >15µm group was 22.4+2.1 µm and in the <15µm group was 9.1+0.4 µm. n = 20. cells per group. Two-sided t-test was used in **d**. ***p<0.001. Source data and exact p values are provided as a Source Data file.

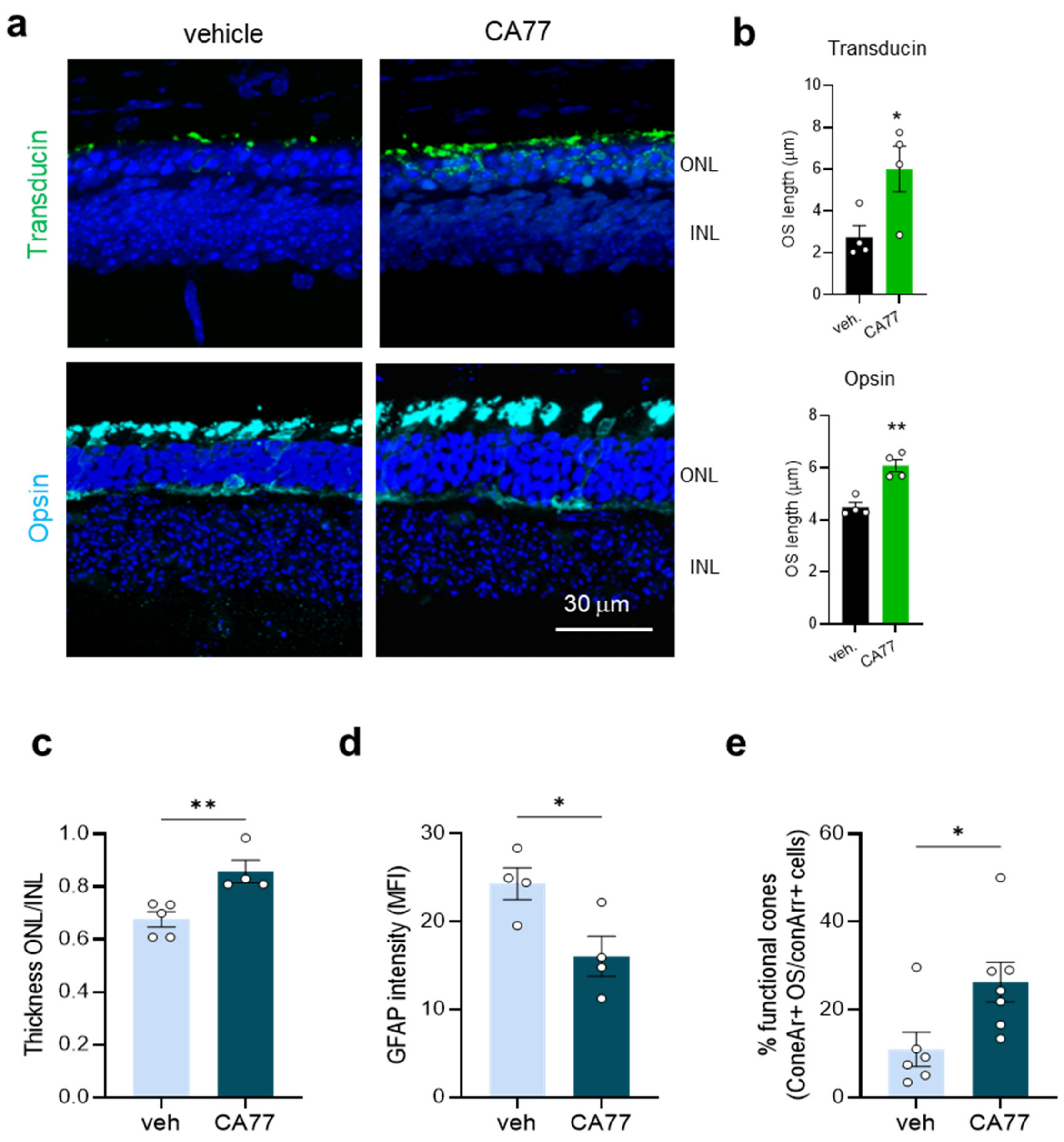

Supplementary Fig. 9. **Effect of CA compounds in rd10 retinas. a, b.** Rod (transducin) and cone (opsin) markers in temporal central retina of rd10 treated from P18 to P25 with daily i.p. injection of vehicle only or 40 mg/kg bw of CA77. n = 8 (vehicle) and 9 (CA treated), from 3 independent experiments. Representative images (**a**) and quantification of outer segment (OS) length (**b**) measured in the whole retina with the markers used in **a**. Nuclei are highlighted with DAPI. n = 4 areas per animal, 4 mice per condition. **c-e.** Ratio of thickness of the outer and inner layers measured in plastic embedded sections after toluidine blue staining (**c**), intensity of GFAP staining (**d**) percentage of functional cones calculated as number of outer segment/number of somas in cone arrestin staining (**e**) of the retinas of rd10 mice 7 days after single intravitreal injection of CA77 (40 µM) at P18. n = 5 veh 4 CA77 (**c**), 4 (**d**), 6 veh 7 CA77 (**e**) Individual values and mean+s.e.m. are shown. Two-sided unpaired t test in c and e and two-sided Mann-Whitney test in d were used. * p<0.05 and **p<0.01. Source data and exact p values are provided as a Source Data file.

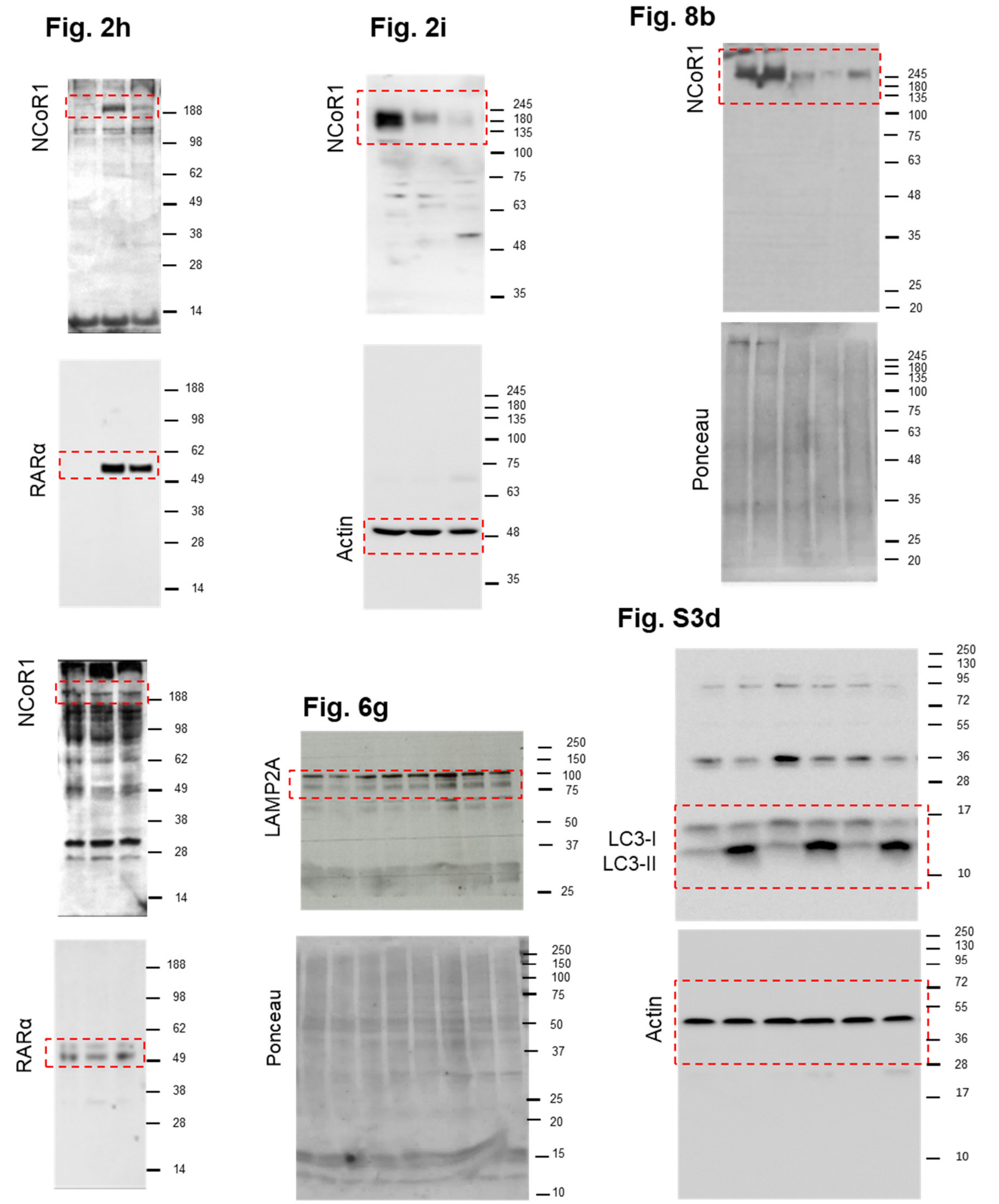

Supplementary Fig. 10. **Uncropped gels from main and supplementary figures.** Uncropped images of the immunoblots shown in main and supplementary figures. The image in the figures is marked with a red doted box. Molecular weight markers (in kDa) are shown on the right

Supplementary Note

Synthesis of 7-chloro-3-(p-tolyl)-2H-benzo[b][1,4]oxazine (AR7):

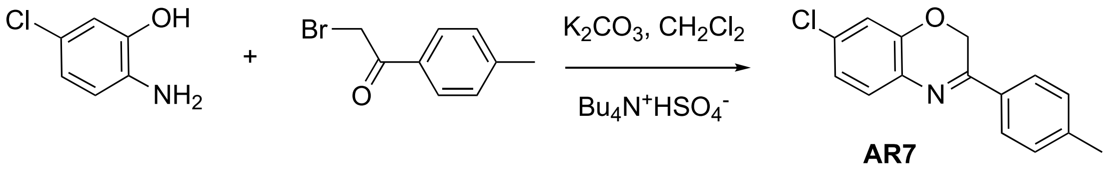

AR7

Preparation of AR7 was as described before [11]. Briefly, 2-bromo-4-chloroacetophenone (0.01 mol) in dichloromethane was added drop-wise to a solution of 2-aminophenol in dichloromethane, aqueous potassium carbonate (20% w/v) and tetrabutylammonium hydrogen sulphate (0.0005 mol). The resulting mixture was refluxed till completion for 4–6 h and the organic layer was extracted with dichloromethane and dried over sodium sulphate evaporated in vacuum to give a crude solid product. The solid was then recrystallized with hot ethanol to obtain pure yield 95%.

AR7 : 1H NMR (300 MHz, $CDCl_3$): δ = 2.44 (s, 3H), 5.09 (s, 2H), 6.91-6.98 (m, 2H), 7.12 (d, J = 12Hz, 2H), 7.33-7.38 (d, J = 8 Hz, 1H), 7.82-7.84 (d, J = 12 Hz, 2H); ^{13}C NMR (75 MHz, $CDCl_3$): δ = 21.56, 62.82, 76.58, 115.94, 122.46, 126.41, 128.34, 132.15, 132.44, 133.02, 141.97, 146.82, 158.94; HRMS for $C_{15}H_{12}ClNO$ ($[M+H]^+$: calculated 258.0641, found: 258.0702.

Synthesis of 3-([1,1'-biphenyl]-4-yl)-7-chloro-2H-benzo[b][1,4]oxazine (CA39):

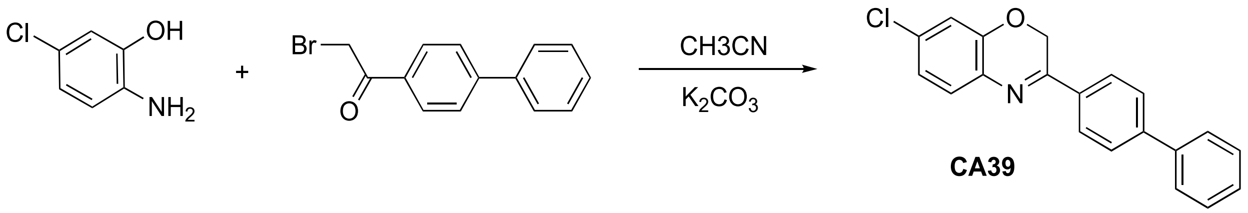

CA39

In a 20mL screw-cap vial with stir bar, 2-amino-5-chlorophenol (1 g, 6.96 mmol) in acetonitrile (40 mL) potassium carbonate (1.92 g, 13.92 mmol) was added. To this mixture 1-([1,1'-biphenyl]-4-yl)-2-bromoethan-1-one (2.15 g, 8.36 mmol) in acetonitrile (20 mL) was added dropwise at room temperature. The reaction mixture was then stirred overnight at room temperature. TLC analysis

of a reaction aliquot (water/EtOAc micro-workup) indicated full conversion. Then the solvent was evaporated and the residue was dissolved in dichloromethane (20 mL). The organic layer was washed with water, brine and dried over Na_2SO_4. The desired compound was isolated through silica gel chromatography (hexanes/ ethyl acetate: 15/1). Recrystallization with isopropanol gave a pale yellow powder (CA39; 1.2 g, 53.9%).

CA39 **:** ^{1}H-NMR (600 MHz, dmso-d_6): δ 8.10 (d, *J* = 8.5 Hz, 2H), 7.84 (d, *J* = 8.5 Hz, 2H), 7.76 (dd, *J* =8.3, 1.1 Hz, 2H), 7.51 (t, *J* = 7.7 Hz, 2H), 7.42 (t, *J* = 7.4 Hz, 1H), 7.39 (d, *J* = 8.1 Hz, 1H), 7.09–7.07 (m, 2H), 5.28 (s, 2H). ^{13}C-NMR (151 MHz, $CDCl_3$): δ 159.3, 147.0, 142.9, 139.0, 133.4, 132.4, 131.8, 129.0, 128.4, 128.1, 127.3, 126.81, 126.73, 122.1, 115.5, 62.5. HRMS for $C_{20}H_{14}ClNO$, M+H : calculated: 320.0837, found: 320.0839

Synthesis of *N*-(4-(7-chloro-2H-benzo[b][1,4]oxazin-3-yl)phenyl)acetamide (CA77):

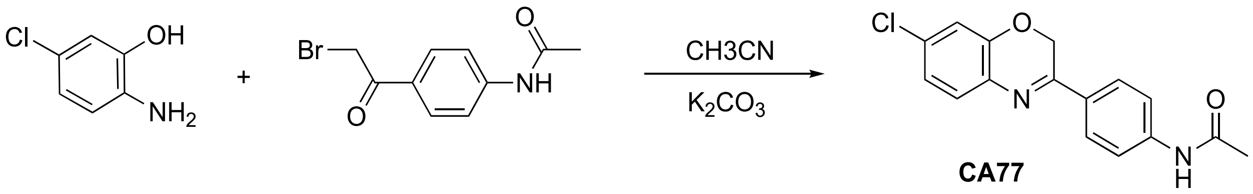

Following a procedure analogous to the procedure described for CA39, using 2-amino-5-chlorophenol (1.02 g, 7.1 mmol) and *N*-(4-(2-bromoacetyl)phenyl)acetamide (2 g, 7.8 mmol) in acetonitrile under reflux overnight. Recrystallization with isopropanol gave the desired compound as white solid (CA77, 560 mg, 26.2%).

CA77 **:** ^{1}H-NMR (600 MHz, dmso-d_6): δ 10.24 (s, 1H), 7.96 (d, *J* = 8.8 Hz, 2H), 7.72 (d, *J* = 8.8 Hz, 2H), 7.33 (d, *J* = 8.8 Hz, 1H), 7.07–7.05 (m, 2H), 5.20 (s, 2H), 2.08 (s, 3H). ^{13}C-NMR (151 MHz, $CDCl_3$): δ 168.8, 159.1, 147.0, 142.4, 132.6, 131.4, 128.9, 128.1, 127.7, 122.1, 118.5, 115.6, 62.4,

24.2. HRMS for $C_{16}H_{13}ClN_2O_2$, M+H: calculated: 301.0738, found: 301.0741.

Synthesis of perfluorophenyl 4-(7-chloro-2*H*-benzo[*b*][1,4]oxazin-3-yl)benzoate.

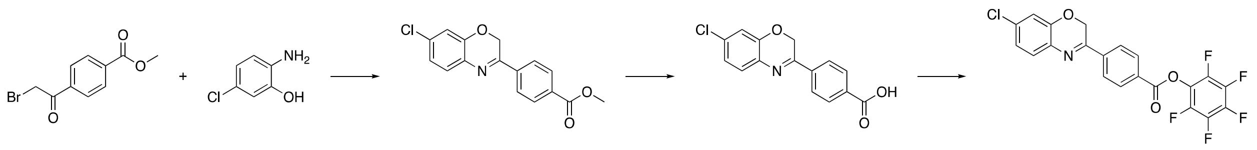

To 2-amino-5-chlorophenol (2.6 g, 10.11 mmol) in dichloromethane (50 mL) was added K_2CO_3 (2.79 g, 20.22 mmol), tetrabutylammonium bisulfate (171.6 mg, 0.5 mmol). Into this, methyl 4-(2-bromoacetyl)benzoate (1.6 mg, 11.12 mmol) in dichloromethane (50 mL) was added dropwise at room temperature. The reaction was then stirred overnight under reflux. The reaction was washed with water, brine and dried over Na_2SO_4. The desired compound was isolated through silica gel chromatography. Recrystallization with hot ethanol gave a pink-yellow powder methyl 4-(7-chloro-2*H*-benzo[*b*][1,4]oxazin-3-yl)benzoate 1.8 g, yield 59%.

^{1}H-NMR (300 MHz, $CDCl_3$): δ 8.14 (d, *J*= 0.02 Hz, 2H), 7.98 (d, *J*= 0.03 Hz, 2H), 7.37 (d, *J*= 0.02 Hz, 1H), 7.02 (d, *J*=0.03 Hz, 1H), 6.93 (s, 1H), 5.08 (s, 2H), 3.95 (s, 3H). HRMS: for $C_{16}H_{12}ClNO_3$ (M+H): calculated 302.0578, found: 302.0580.

Then methyl 4-(7-chloro-2*H*-benzo[*b*][1,4]oxazin-3-yl)benzoate (1.5 g, 4.97 mmol) was stirred with 1 N LiOH (aq). (49.7 mmol, 1.19g) in 20 mL THF at room temperature for 4 hours. THF was removed under reduced pressure followed by slow addition of 4 N HCl and a white solid 4-(7-chloro-2*H*-benzo[*b*][1,4]oxazin-3-yl)benzoic acid precipitated. The precipitate was filtered and washed with cold EtOH and dried in air (quantitative yield).

^{1}H-NMR (300 MHz, dmso-d6): δ 13.23 (s, 1H), 8.04-8.13 (m, 4H), 7.42 (d, *J*= 0.03 Hz, 1H), 7.10 (d, *J*= 0.01 Hz, 2H), 5.29 (s, 2H). HRMS for $C_{15}H_{10}ClNO_3$ (M+H): calculated 288.0422, found: 288.0421.

Without further purification, 4-(7-chloro-2*H*-benzo[*b*][1,4]oxazin-3-yl)benzoic acid (1 g, 3.47 mmol) reacts with pentafluorophenyl trifluoroacetate (1 mL, 1.65 g, 5.9 mmol), triethylamine (0.84 mL, 6.07 mmol) in dry DMF under N_2 at room temperature. Ethyl acetate (40 mL) was added to dilute the reaction mixture which was washed with water, brine and dried over Na_2SO_4. The desired compound was isolated through silica gel chromatography. A yellow solid perfluorophenyl 4-(7-chloro-2*H*-benzo[*b*][1,4]oxazin-3-yl)benzoate was obtained 1.18g, yield 74.9%.

^{1}H-NMR (300 MHz, dmso-d6): δ ppm: 8.24-8.34 (m, *J*= 0.03 Hz, 4H), 7.43 (d, *J*= 0.03 Hz, 1H), 7.11-7.13 (d, *J*= 0.02 Hz, 2H), 5.35 (s, 2H), HRMS for $C_{21}H_9ClF_5NO_3$ (M+H): calculated 454.0264, found: 454.0271.

Synthesis of 4-(7-chloro-2*H*-benzo[*b*][1,4]oxazin-3-yl)-*N*-(15-oxo-19-((4*S*)-2-oxohexahydro-1*H*-thieno[3,4-*d*]imidazol-4-yl)-4,7,10-trioxa-14-azanonadecyl)benzamide (Biotin-CA)

Biotin-CA

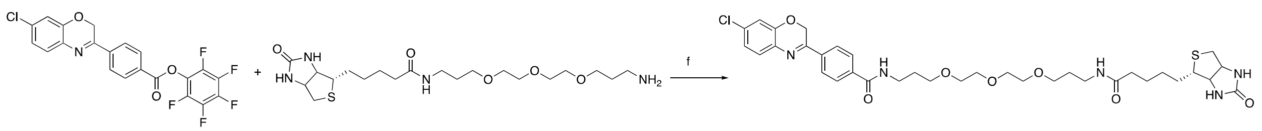

To Biotin-PEG-amine (*N*-(3-(2-(2-(3-aminopropoxy)ethoxy)ethoxy)propyl)-5-((4*S*)-2-oxohexa hydro-1*H*-thieno[3,4-*d*]imidazol-4-yl)pentanamide (0.98 g, 2.2 mmol) in dry DMF (15 mL) was added triethylamine (0.61mL, 4.4 mmol) under N_2. Then perfluorophenyl 4-(7-chloro-2*H*-benzo[*b*][1,4]oxazin-3-yl)benzoate (1 g, 2.2 mmol) in dry dichloromethane (25 mL) was added

dropwise. The mixture was stirred at room temperature overnight. All solvent was removed under reduced pressure to give a dark oily mixture which was washed with ethyl acetate, ethyl acetate/methanol (10/1). The residue was purified on silica gel (ethyl acetate/ methanol, 10/1, with 1% $NH_3 \cdot H_2O$) to provide a yellow solid 0.91 g, yield 58.1%.

Biotin-CA: ^{1}H-NMR (600 MHz, dmso-d_6): δ 8.59 (d, *J*= 0.01 Hz, 1 H), 8.07 (d, *J*= 0.01 Hz, 2 H), 7.96 (d, *J*= 0.01 Hz, 2 H), 7.73 (s, 1 H), 7.39 (d, *J*= 0.01 Hz, 1 H), 7.08 (m, 1 H), 6.41 (s, 1 H), 6.35 (s, 1 H), 5.28 (s, 2 H), 4.29 (t, *J*= 0.01 Hz, 1 H), 4.10 (t, *J*= 0.01 Hz, 1 H), 3.46-3.54 (m, 11 H), 3.32-3.39 (m, 5H), 3.07 (m, 3 H), 2.80 (dd, *J*1= 0.01, *J*2= 0.02 Hz, 1H), 2.56 (d, *J*=0.02, 1H), 2.03 (t, *J*= 0.01 Hz, 2 H), 1.78 (m, 2 H), 1.60 (m, 3 H), 1.49 (m, 3 H), 1.28 (m, 3 H). HRMS for $C_{35}H_{46}ClN_5O_7S$ (M+H): calculated 716.2879, found: 716.2887.

Supplementary Table 1. CA-mediated transcriptional changes.

Gene Identifier	Gene Symbol	Log2 scale			P-value (Differentially)			Direction	P<0.01
		AR7	CA39	CA77	AR7	CA39	CA77		
NM_019465	Crtam	0.0295	0.02443	0.02383	8.5E-05	7.8E-05	7.6E-05	Down	With the 3 compounds
NM_152802	Defb12	0.01552	0.01369	0.01656	0.00137	0.00139	0.0014	Down	
chr13:64044376-64045368_R	Non-coding	0.17775	0.15702	0.1362	0.00255	0.00193	0.00142	Down	
AY170623	Dsg4	0.04227	0.04187	0.02136	0.00231	0.00241	0.00176	Down	
NM_007941	Stx2	0.06594	0.11796	0.09232	0.00118	0.00263	0.00182	Down	
chr12:52944867-52971992_R	Non-coding	0.05586	0.02965	0.03376	0.00386	0.00292	0.00298	Down	
NM_010482	Htr1b	0.02601	0.02191	0.04553	0.0032	0.0032	0.00416	Down	
chr16:44717531-44724206_F	Non-coding	0.02202	0.01733	0.03891	0.00435	0.00435	0.0054	Down	
chr5:54053328-54053870_F	Non-coding	0.08925	0.08567	0.08612	0.00448	0.00452	0.0045	Down	
NR_024017	4930594C11Rik	0.02681	0.02758	0.0118	0.00487	0.0052	0.00413	Down	
chr6:36765550-36793825_F	Non-coding	0.04136	0.01728	0.01829	0.00654	0.0053	0.00512	Down	
ENSMUST00000097448	Anti-sense	0.03891	0.03251	0.02606	0.00671	0.00663	0.00591	Down	
chr1:64232564-64349617_R	Non-coding	0.03837	0.044	0.03926	0.00658	0.00742	0.00678	Down	
NM_010446	Foxa2	0.06131	0.05303	0.03773	0.009	0.00877	0.00715	Down	
chr15:83266264-83294664_F	Non-coding	0.05281	0.07415	0.06682	0.00758	0.01011	0.00909	Down	AR7, A77
NM_008706	Nqo1	4.84289	1.29983	4.96787	0.00024	0.44799	0.00023	Up	
NM_010357	Gsta4	26.5846	2.33075	18.7705	2.5E-06	0.038	4.9E-06	Up	
NM_001122668	E330014E10Rik	26.1281	1.58381	33.8974	0.00483	0.82855	0.00427	Up	
CK334688	AU017674	7.51339	18.0101	22.5451	0.06589	0.00596	0.00379	Up	CA39, CA77
chr1:138570107-138632310_F	Non-coding	1.40947	7.86325	6.31921	0.49282	0.0002	0.00067	Up	
chr1:138570107-138632310_F	Non-coding	1.40947	7.86325	6.31921	0.49282	0.0002	0.00067	Up	
chr16:39984031-40081556_R	Non-coding	0.54799	0.39078	0.07815	0.04968	0.00497	5.3E-06	Down	
XR_168418	Gm5106	0.42622	0.28915	0.09934	0.01916	0.00244	6.1E-05	Down	
NM_181418	Ushbp1	0.21988	0.18982	0.16476	0.01199	0.00887	0.00665	Down	
chr11:70196232-70203307_R	Non-coding	0.18851	0.13334	0.14295	0.01035	0.00566	0.00642	Down	
NM_178689	Eno4	0.1098	0.10788	0.23172	0.00575	0.00594	0.02332	Down	AR7, CA77

Ratio of expression relative to untreated cells (in Log2 scale) and p values of genes showing significant changes in expression upon treatment of NIH3T3 cells with AR7, CA39 or CA77 (for details see Fig 2d and Methods). Genes are grouped according to the number of compounds for which they show significantly different expression than untreated cells (indicated under p<0.01, blue column). Upregulation or downregulated expression is indicated as Up or Down, respectively under direction. Two-sided unpaired t-test was used for comparison with untrated.

Supplementary Table 2. Sequence of forward (F) and reverse (R) primers used for qPCR

Lamp2a	F-5'-GCAGTGCAGATGAAGACAAC-3'<br>R-5'-AGTATGATGGCGCTTGAGAC-3'
β-actin	*F-5'-AA GGACTCCTA TAGTGGG TGACGA-3'*<br>*R-5'-ATCTTCTCCATGTCGTCCCAGTTG-3'*
PHLPP1	F-5'-AGCTTCCCGGCAAGTA AAAT-3'<br>R-5'-GGTGGCAGGTTTTCTGGTAA-3'
Dendra2	F-5'-AAGGGCATCTGCACCATCCG-3'<br>R-5'-CGTGCTCG TACAGCTTCACCTTG-3'
GFAP	F-5'-CGGAGACGCATCACCTCTG-3',<br>R-5'-TGGAGGAGTCATTCGAGACAA-3'
HSC70	F-5'-TCTCGGCACCACCTACTCC-3'<br>R-5'-CCCGCATCAGACGTTTGGCA-3'
HSP40	F-5'-TTCGACCGCTATGGAGAGGAA-3'<br>R-5'-CACCGAAGAACTCAGCAAACA-3'
HSP90AA1	F-5'-GACGCTCTGGATAAAATCCGTT-3'<br>R-5'-TGGGAATGAGATTGATGTGCAG-3'
HSP90AB1	F-5'-AAACAAGGAGATTTTCCTCCGC-3'<br>R-5'-CCGTCAGGCTCTCATATCGAAT-3'
NCOR1	F-5'-CCCATTTCCACCGTGTTAGTG-3'<br>R-5'-AAGGTGAAGCATTTTGGTCATCT-3'
RAC1	F-5'-ACGGAGCTGTTGGTAAAACCT-3'<br>R-5'-AGACGGTGGGGATGTACTCTC-3'
RARa	F-5'-CTAGCCGATTTGCACGGATG-3'<br>R-5'-GGGTCTCGATGGAGTGGTTT-3'
AKT1	F-5'-ATCCCCTCCAACAACTTCTCAGT-3'<br>R-5'-CCTTCCCGTCCACTCTTCTCTTTC-3'
AKT2	F-5'-ACGTGGTGAATACATCAAGACC-3'<br>R-5'-GGGCCTCTCCTTATACCCAAT-3'
CTSA	F-5'-CAGCCCTCTTTCCGGCAATA-3'<br>R-5'-TTTGGGTCGTTCTGCGACTC-3'
NFATc1	F-5'-GGAGAGTCCGAGAATCGAGAT-3'<br>R-5'-TTGCAGCTAGGAAGTACGTCT-3'
NFe2L2	F-5'-CTTTAGTCAGCGACAGAAGGAC-3'<br>R-5'-AGGCATCTTGTTTGGGAATGTG-3'
Rab11a	F-5'-AGGAGCGGTACAGGGCTATAA-3'<br>R-5'-ATGTGAGATGCTTAGCAATGTCA-3'
LAMP1	F-5'-TAGTGCCCACATTCAGCATCTCCA-3'<br>R-5'-TTCCACAGACCCAAACCTGTCACT-3'
LAMP2B	F-5'-GGTGCTGGTCTTTCAGGCTTGATT-3'<br>R-5'-ACCACCCAATCTAAGAGCAGGACT-3'
LAMP2C	F-5'-ATGTGCTGCTGACTCTGACCTCAA-3'<br>R-5'-TGGAAGCACGAGACTGGCTTGATT-3'
RICTOR	F-5'-GCTGCGCTATCTCATCCAAGA-3'<br>R-5'-GGGTTCTGAAGTGCTAGTTCAC-3'
B2M	F-5'-CACTGAATTCACCCCCACTGA-3'<br>R-5'-TCACATGTCTCGATCCCAGT-3'
TBP	F-5'-GAAGCTGCGGTACAATTCCAG-3'<br>R-5'-CCCCTTGTACCCTTCACCSAAT-3'
EEF1A1	F-5'-GAAGATCGGCTACAACCCAG-3'<br>R-5'-CTTCCTTACGCTCTACTTTCCAG-3'
