# Supplementary material for: Targeting retinoic acid receptor alpha-corepressor interaction activates chaperone-mediated autophagy and protects against retinal degeneration
Source: Nat Commun. 2022 Jul 21;13:4220. doi: 10.1038/s41467-022-31869-1 (PMC9304322; doi:10.1038/s41467-022-31869-1)
Supplement: Supplementary file 2 — Reporting Summary [file 41467_2022_31869_MOESM2_ESM.pdf]

nature portfolio

Corresponding author(s): Patricia Boya, Evridipidis Gavathiotis, Ana Maria Cuervo (contact corresponding)

Last updated by author(s): Jun 8, 2022

Reporting Summary

Nature Portfolio wishes to improve the reproducibility of the work that we publish. This form provides structure for consistency and transparency in reporting. For further information on Nature Portfolio policies, see our Editorial Policies and the Editorial Policy Checklist.

Statistics

For all statistical analyses, confirm that the following items are present in the figure legend, table legend, main text, or Methods section.

n/a	Confirmed	
☐	☒	The exact sample size (*n*) for each experimental group/condition, given as a discrete number and unit of measurement
☐	☒	A statement on whether measurements were taken from distinct samples or whether the same sample was measured repeatedly
☐	☒	The statistical test(s) used AND whether they are one- or two-sided<br>*Only common tests should be described solely by name; describe more complex techniques in the Methods section.*
☒	☐	A description of all covariates tested
☐	☒	A description of any assumptions or corrections, such as tests of normality and adjustment for multiple comparisons
☐	☒	A full description of the statistical parameters including central tendency (e.g. means) or other basic estimates (e.g. regression coefficient) AND variation (e.g. standard deviation) or associated estimates of uncertainty (e.g. confidence intervals)
☐	☒	For null hypothesis testing, the test statistic (e.g. *F*, *t*, *r*) with confidence intervals, effect sizes, degrees of freedom and *P* value noted<br>*Give P values as exact values whenever suitable.*
☒	☐	For Bayesian analysis, information on the choice of priors and Markov chain Monte Carlo settings
☒	☐	For hierarchical and complex designs, identification of the appropriate level for tests and full reporting of outcomes
☒	☐	Estimates of effect sizes (e.g. Cohen's *d*, Pearson's *r*), indicating how they were calculated

Our web collection on statistics for biologists contains articles on many of the points above.

Software and code

Policy information about availability of computer code

Data collection	Softwares for data collection are listed in the corresponding sections of the Methods. Leica LAS-X (3.7.4.23463) was used for image acquisition. Modeling data were collected with Schrodinger software suite (Releases 2016-2020, Schrödinger, LLC) and Pymol (Version 2.3, The PyMOL Molecular Graphics System, Schrödinger, LLC).
Data analysis	All software packages used in this study are listed in the "Statistical Analysis" section of the Methods . Basic data handling was done in Microsolft Excel 365 (v.16.57 and v.2101). G power analysis was performed with the G*Power software (v.3.1.9.7, RRID:SCR_013726). Data analysis was performed with Prism software (v9 - Graph Pad Software Inc). Image analysis and quantification was performed using ImageJ (v.2.1.0). Immunoblot quantification with Quantity One software (v.4.6.8., Bio-Rad). Structural and small molecule data were analyzed with modules GLIDE, EPIK, LIGPREP, QIKPROP, MAESTRO of Schrodinger software suite (Releases 2016-2021).

For manuscripts utilizing custom algorithms or software that are central to the research but not yet described in published literature, software must be made available to editors and reviewers. We strongly encourage code deposition in a community repository (e.g. GitHub). See the Nature Portfolio guidelines for submitting code & software for further information.

Data

Policy information about availability of data

All manuscripts must include a data availability statement. This statement should provide the following information, where applicable:

- Accession codes, unique identifiers, or web links for publicly available datasets
- A description of any restrictions on data availability
- For clinical datasets or third party data, please ensure that the statement adheres to our policy

There are not restrictions on data availability in this manuscript. The data in main and supplementary figures generated in this study are provide in the

Supplementary Information/Source Data file as an Excel worksheet organized by figures and inside each figure sheet by panels and it includes raw data and full statistic report. Transcriptomic data has been deposited in the Gene Expression Omnibus (GEO) database under accession code GSE194291 [https://www.ncbi.nlm.nih.gov/geo/query/acc.cgi?acc=GSE194291] and GSE194292 [https://www.ncbi.nlm.nih.gov/geo/query/acc.cgi?acc=GSE194292]. The structural data used in this study are available in the PDB protein data bank under accession code 1DKF [https://www.rcsb.org/structure/1dkf] and 3KMZ [https://www.rcsb.org/structure/3KMZ].

Field-specific reporting

Please select the one below that is the best fit for your research. If you are not sure, read the appropriate sections before making your selection.

☒ Life sciences ☐ Behavioural & social sciences ☐ Ecological, evolutionary & environmental sciences

For a reference copy of the document with all sections, see nature.com/documents/nr-reporting-summary-flat.pdf

Life sciences study design

All studies must disclose on these points even when the disclosure is negative.

Sample size	The number of animals used per experiment was calculated through power analysis with the G*Power software (v.3.1.9.7, RRID:SCR_013726) based on previous results for a significance of 0.05 and a statistical power of 0.8. For the studies involving cells in culture treatment groups were attributed randomly between wells and plates to account for well or tube positioning effects. We determine number of experimental repetitions to account for technical variability and changes in culture conditions based on our previous studies using those systems including both studies showing up regulation and down regulation of CMA, increase and decrease of protein degradation and decrease of cell viability . For examples of previous studies used to calculate number of experiments with cells see PMID: 32005807 ; PMID: 33854069; PMID: 33891876 ; PMID: 34876687. For molecular docking studies sample size was chosen based on previous experience with similar studies PMID 30718816 ; PMID: 33602934.
Data exclusions	No mouse was excluded from the analysis unless there was technical reason, accident or the mouse was determined to be in very poor health by the veterinarian, which did not occurred as part of this study. Technical reasons were predefined before the study and included involuntary puncture of an abdominal organ by the syringe or inability to administer drug/vehicle because of anatomic abnormality or signs of bulgy abdomen/ascytis. Accidents were also predefined before initiating the study and included severe bitting by another litermate. Technical reasons or accidents did not require to eliminate any of the animals.
Replication	Every experiment was performed in at least 3 independent replicates. Experiments in cells in culture were performed on different days to confirm reproducibility of the procedures. All independent replications were successful.
Randomization	Animals were randomly attributed to each treatment group using the "SELECT BETWEEN RANGE" function in Microsoft Excel. For the studies involving cells in culture treatment groups were attributed randomly between wells and plates to account for well or tube positioning effects
Blinding	Investigators were blinded to the treatment during data collection and analysis for both in vivo and in vitro experiments and unblinding was done when the analysis was completed for plotting and image composition of the figures.

Reporting for specific materials, systems and methods

We require information from authors about some types of materials, experimental systems and methods used in many studies. Here, indicate whether each material, system or method listed is relevant to your study. If you are not sure if a list item applies to your research, read the appropriate section before selecting a response.

Materials & experimental systems

n/a	Involved in the study	
☐	☒	Antibodies
☐	☒	Eukaryotic cell lines
☒	☐	Palaeontology and archaeology
☐	☒	Animals and other organisms
☐	☒	Human research participants
☒	☐	Clinical data
☒	☐	Dual use research of concern

Methods

n/a	Involved in the study	
☒	☐	ChIP-seq
☒	☐	Flow cytometry
☒	☐	MRI-based neuroimaging

Antibodies

Antibodies used	Primary antibodies were from the following sources (dilution for use in immunoblot (IB) or immunofluorescence (IF) and clone indicated in brackets): rabbit anti LC3B (1/1000 IB, MBL pm036), mouse anti β-actin (1/10000 IB, Sigma, A4700, clone AC-40), chicken anti MAP2 (1/2000 IF, Biolegend, 822501), mouse anti GFAP (1/1000 IF, Millipore, MAB360, clone GA5), rabbit anti GFAP (1/500 IF, DAKO, Z0334), mouse anti S100 (1/1000 IF, Abcam, ab7852, clone B32.1), goat anti Iba1 (1/100 IF, Abcam, ab5076), rabbit anti RARα (1/1000 IF and IB, Cell Signaling, 2554), mouse anti visual (rod) arrestin (1/200 IF, Santacruz Biotechnologies, C-3, Sc-166383), rabbit anti Opsin R/G (1/1000 IF, Millipore, AB5405), rabbit anti transducin (1/200 IF, Santacruz Biotechnologies, sc-389),

	rabbit anti cone arrestin (1/1000 IF, Millipore, AB15282), rabbit anti NCoR1 (1/100 IF and IB, Cell Signaling, 5948) and rabbit anti L2A (1/2000 IB, Invitrogen, 51-2200).<br>Secondary antidoes were from the following sources (dilution for use and clone indicated in brackets): Anti-mouse IgG secondary antibody Alexa Fluor 568 (1/500 IF, Invitrogen, A-11004), Anti-mouse IgG secondary antibody Alexa Fluor 647 (1/500 IF, Invitrogen, A-32728), Anti-goat IgG secondary antibody, Alexa Fluor 594 (1/500 IF, Invitrogen, A-11012), Anti-rabbit IgG secondary antibody, Alexa Fluor 568 (1/500 IF Invitrogen, A-11011), Anti-chicken IgY secondary antibody, Alexa Fluor 488 (1/500 IF, Invitrogen, A-11039), Anti-rabbit secondary antibody, HRP (1:5000 IB, ThermoFisher, 31460), Anti-mouse secondary antibody, HRP (1:5000 IB, ThermoFisher, 31430)
Validation	All antibodies used in this study were from commercial sources and details on their validation can be found using the catalogue number provided above. Once received in our laboratories we determine the optimal conditions for our experiments using the multiple dilution method to determine the higher dilution that was giving a signal in order to minimize detection of unspecific signals. In the case of antibodies against LAMP2A, RARa and NCoR1 we used knock-down cell lines for validation of the antibodies.

Eukaryotic cell lines

Policy information about cell lines

Cell line source(s)	NIH3T3 mouse fibroblasts, CaCo2 cells and the N2a neuroblastoma cell line were obtained from the American Type Culture Collection. Primary human fibroblasts (GM01651) were from Coriell Repository.
Authentication	All cells were validated by genomic PCR
Mycoplasma contamination	All the cell lines were tested for mycoplasma contamination using DNA staining protocol with Hoechst 33258 dye and all of them tested negative.
Commonly misidentified lines<br>(See ICLAC register)	None of the cells used in this study are commonly misidentified lines.

Animals and other organisms

Policy information about studies involving animals; ARRIVE guidelines recommended for reporting animal research

Laboratory animals	C57BL/6J mice wild type and homozygous for the Pde6 mutation (rd10 mice) both male and female were used a the specific post-natal ages indicated in each experiment.<br>C7BL/6J KFERQ-Dendra mice males and females were used at 4 months of age.<br>ICR (CD-1) outbred 3 months old male mice were used of pharmacokinetic studies.
Wild animals	No wild animals were used in the study
Field-collected samples	No field-collected samples were used in the study
Ethics oversight	All animal studies and procedures complied with ethical regulations, were performed in accordance with the European Union guidelines and the ARVO Statement for the Use of Animals in Ophthalmic and Vision Research and were approved by the Institutional Animal Care and Use Committee at the Albert Einstein College of Medicine, the CSIC Bioetica Comite and approved by the Comunidad de Madrid, PROEX232/17.

Note that full information on the approval of the study protocol must also be provided in the manuscript.

Human research participants

Policy information about studies involving human research participants

Population characteristics	Sources of the human tissues for the generation of the retinal organoids are detailed in the original studies24, 25. Briefly, in24 patient mononuclear cells were collected and subjected to a plasmid-based reprogramming system to generate human iPSCs that were subsequently subjected to protocols for trilineage differentiation. In25, consent skin biopsies were obtained to isolate dermal fibroblasts and iPSCs were generated from two unrelated RP patients and 2 controls.
Recruitment	Data from human tissue from healthy individuals and retinitis pigmentosa patients was obtained from previous published studies and no recruitment or collection of human tissue was performed for the completion of this work.
Ethics oversight	The ethics committees that approved the published studies from where we obtained the transcriptional data: In both studies, collection of human samples was approved by the research ethics committees in their respective institutions (The Eye Hospital of Wenzhou Medical University Ethics Committee24 and The Moorfields Eye Hospital and Royal Victoria Eye and Ear Hospital Dublin Research Ethics Committees25). The patients/participants provided their written informed consent to participate in those studies.

Note that full information on the approval of the study protocol must also be provided in the manuscript.
